# Evidence for a semisolid phase state of aerosols and droplets relevant to the airborne and surface survival of pathogens

**DOI:** 10.1073/pnas.2109750119

**Published:** 2022-01-21

**Authors:** Erik Huynh, Anna Olinger, David Woolley, Ravleen Kaur Kohli, Jack M. Choczynski, James F. Davies, Kaisen Lin, Linsey C. Marr, Ryan D. Davis

**Affiliations:** ^a^Department of Chemistry, Trinity University, San Antonio, TX 78212;; ^b^Department of Chemistry, University of California, Riverside, CA 92521;; ^c^Department of Civil and Environmental Engineering, Virginia Tech, Blacksburg, VA 24061

**Keywords:** disease transmission, viral survival, aerosol-phase state, pathogens, amorphous phases

## Abstract

The phase state of respiratory aerosols and droplets has been linked to the humidity-dependent survival of pathogens such as SARS-CoV-2. To inform strategies to mitigate the spread of infectious disease, it is thus necessary to understand the humidity-dependent phase changes associated with the particles in which pathogens are suspended. Here, we study phase changes of levitated aerosols and droplets composed of model respiratory compounds (salt and protein) and growth media (organic–inorganic mixtures commonly used in studies of pathogen survival) with decreasing relative humidity (RH). Efflorescence was suppressed in many particle compositions and thus unlikely to fully account for the humidity-dependent survival of viruses. Rather, we identify organic-based, semisolid phase states that form under equilibrium conditions at intermediate RH (45 to 80%). A higher-protein content causes particles to exist in a semisolid state under a wider range of RH conditions. Diffusion and, thus, disinfection kinetics are expected to be inhibited in these semisolid states. These observations suggest that organic-based, semisolid states are an important consideration to account for the recovery of virus viability at low RH observed in previous studies. We propose a mechanism in which the semisolid phase shields pathogens from inactivation by hindering the diffusion of solutes. This suggests that the exogenous lifetime of pathogens will depend, in part, on the organic composition of the carrier respiratory particle and thus its origin in the respiratory tract. Furthermore, this work highlights the importance of accounting for spatial heterogeneities and time-dependent changes in the properties of aerosols and droplets undergoing evaporation in studies of pathogen viability.

## Significance

Ambient humidity can influence the survival of pathogens in respiratory aerosols and droplets, although the mechanism and optimum humidity level for public health remain unclear. Here, we present evidence for a humidity-dependent, semisolid state of aerosols and droplets relevant to pathogen survival. These observations indicate that a semisolid state may protect pathogens from inactivation by hindering disinfection reactions at intermediate-to-low humidity levels. The formation of the semisolid state was dependent on the composition of the aerosols, which suggests that the humidity for optimum pathogen destruction will depend on the composition of respiratory particles released from an infected host. These observations can be used to help interpret laboratory studies and inform public health recommendations.

Pathogenic organisms, including bacteria and viruses, are responsible for the global spread of many diseases, such as COVID-19 and seasonal influenza. Transmission of these infectious diseases is predicated on the transfer of a sufficient number of viable pathogens from one host to another (1–3). Coughing, talking, breathing, and singing by an infected host can release respiratory particles carrying pathogens that can infect via aerosols or droplets or indirectly via fomites that have been contaminated with respiratory emissions (2, 4–6). To trigger an infection, pathogens must remain viable. The mechanisms that control pathogen viability in the environment remain unclear but have been linked to many factors, including the identity of the pathogen, the composition of the medium in which it is suspended (e.g., pH and protein content), exposure to sunlight, ambient temperature, and ambient humidity (1–3, 7–10). The role of humidity is particularly complex since it can simultaneously influence the composition and phase state of respiratory aerosols and droplets (7), and many studies suggest humidity variations can explain the seasonality of, for example, influenza transmission (11). A deeper understanding of how humidity influences pathogen viability is thus critical to understanding and mitigating disease transmission.

Bacteria and viruses in aerosols and droplets tend to respond differently to changes in relative humidity (RH), with bacteria viability continuously decreasing as RH decreases, whereas, for some viruses, viability reaches a minimum at intermediate RH before increasing again at lower RH (that is, viruses often exhibit a U-shaped, RH-dependent viability) (3, 7, 8, 12–16). This U-shaped viability has been established with many viruses, including SARS-CoV-2, MS2, phi-6, influenza A, rhinovirus, and others. Although this trend has been observed for most viruses, there are some exceptions. For example, the viability of equine herpesvirus type 1 has been observed to decrease monotonically with decreasing RH (17). The extent to which viability decreases and the RH at which viral viability recovers when a U-shape is observed depends on the virus (e.g., enveloped or nonenveloped) and the composition of the suspension medium. For example, Lin et al. (3) systematically demonstrated that the inclusion of bovine serum albumin can preserve the viability of both MS2 and phi-6 in droplets but that the viability of MS2 was generally higher than phi-6 at all RH. Differences in the composition of the medium can also explain discrepancies in results between studies on the same virus (2, 3, 8). For example, Yang et al. (8) studied the viability of influenza A in droplets composed of differing suspension media, including growth media and mucus, and reported a U-shaped RH dependence in all cases. However, the extent of inactivation at high RH, and the RH at which viability began to increase, was very dependent on droplet composition. The mechanism for this U-shaped RH dependence, and the interplay between humidity and aerosol and droplet composition, remains unclear.

Previous studies have suggested that RH influences pathogen viability by altering the composition or phase of the surrounding medium. When respiratory particles are emitted, whether they remain airborne or are deposited onto a surface, they experience a drastic decrease in RH moving from the respiratory system (∼100% RH) to ambient RH (<70% RH, typically) (18). Respiratory particles are composed of water in addition to solutes, such as salt and protein, and this decrease in RH drives the evaporation of water to establish an equilibrium between the particle and surrounding air. At equilibrium, the particle-phase water activity (*a_w_*) is equivalent to the gas-phase RH/100. As water evaporates, the nonvolatile solutes (salts, proteins, and surfactants) become highly concentrated (18). For example, in an aqueous NaCl particle at equilibrium with 75% RH (*a_w_* = 0.75), the NaCl concentration is equivalent to a saturated brine solution (19). There is a general consensus that the initial decrease in pathogen viability with decreasing RH is likely due to this increasing solute (disinfectant) concentration (7, 10). The reason for the recovery in viral viability at lower RH is less clear, but it has been suggested that aerosol and droplet-phase changes may be responsible (10, 14).

Changes in ambient RH can induce phase changes as solutes become increasingly supersaturated with dehumidification (8, 10). For example, supersaturated, aqueous NaCl particles have a high probability of efflorescence (crystallization with loss of particle-phase water) when at equilibrium with ∼45% RH (i.e., when *a_w_* ∼0.45) equivalent to a NaCl concentration of ∼12 mol/kg (20), although efflorescence has a finite probability of occurring across a range of RH (21) (see *SI Appendix*, *Factors Influencing Phase Changes* for additional discussion). Pathogens are typically suspended in complex organic–inorganic media (e.g., culture media and respiratory fluid). The phase changes associated with organic–inorganic aerosols are far more varied than for simple, aqueous inorganic aerosols (22–31). With decreasing RH, homogeneously mixed organic–inorganic liquids can transition to an amorphous, glassy, ultraviscous, or gelatinous state in which diffusion and efflorescence are hindered (22–26). In these phase states, hindered diffusion of water can inhibit evaporation and delay the establishment of equilibrium (i.e., *a_w_* and RH are not necessarily equivalent). Under such nonequilibrium conditions, there can be significant spatial gradients in particle-phase viscosity and water content, dependent on the particle’s composition, size, and rate of initial evaporation (23, 32). Spatial heterogeneities are also possible because of liquid–liquid phase separation (e.g., amorphous organic shell and aqueous inorganic core), aggregation (e.g., of organic compounds), or partial efflorescence (e.g., amorphous organic coating of a crystalline inorganic core) (29, 30). Equilibrium phase states are not established instantaneously upon exposure to ambient air because the evaporation of water, and associated increase in solute concentration, is a mass transport process with a finite timescale dependent on particle size, composition, and ambient RH (10, 32–34). During the nonequilibrium evaporation period, *a_w_* is continuously evolving until equilibrium is achieved and significant gradients in viscosity and solute concentration can emerge. Furthermore, in complex chemical systems, multiple phase states can exist simultaneously, with each phase state representing a unique microenvironment that can influence pathogen viability (18). Thus, a detailed understanding of phase changes, including both their spatial and temporal evolution, is likely necessary to develop a mechanistic understanding of humidity-dependent disease transmission. However, no comprehensive understanding of phase states relevant to pathogen viability exists, and many aspects of disease transmission thus remain unclear.

Here, we provide a detailed exploration of the RH-dependent phase state of levitated aerosol relevant to understanding the survival of pathogens and examine the morphology of evaporating droplets on a surface. To clarify the terminology used in this article, “particles” refers to aerosols and droplets generally, “aerosols” are particles that are suspended in a gas, and “droplets” are particles that fall to a surface under the force of gravity. In laboratory studies of pathogen viability, a growth medium is often used as the suspension medium (8, 10). Thus, to facilitate the interpretation of previous studies, we studied particles composed of several growth media: Dulbecco’s modified Eagle’s medium (DMEM), lysogeny broth (LB)—Miller’s modification—and tryptic soy broth (TSB). Furthermore, since real-world transmission will occur via respiratory particles, we also studied the phase state of model respiratory compounds (salts and protein) with variable salt and protein content. Our observations can thus be used to interpret previous findings as well as make qualitative predictions for survival of pathogens in aerosols and droplets under ambient conditions.

## Results

### Identification of Crystalline and Amorphous Phase States of Levitated Aerosol.

**Fig. 1.**
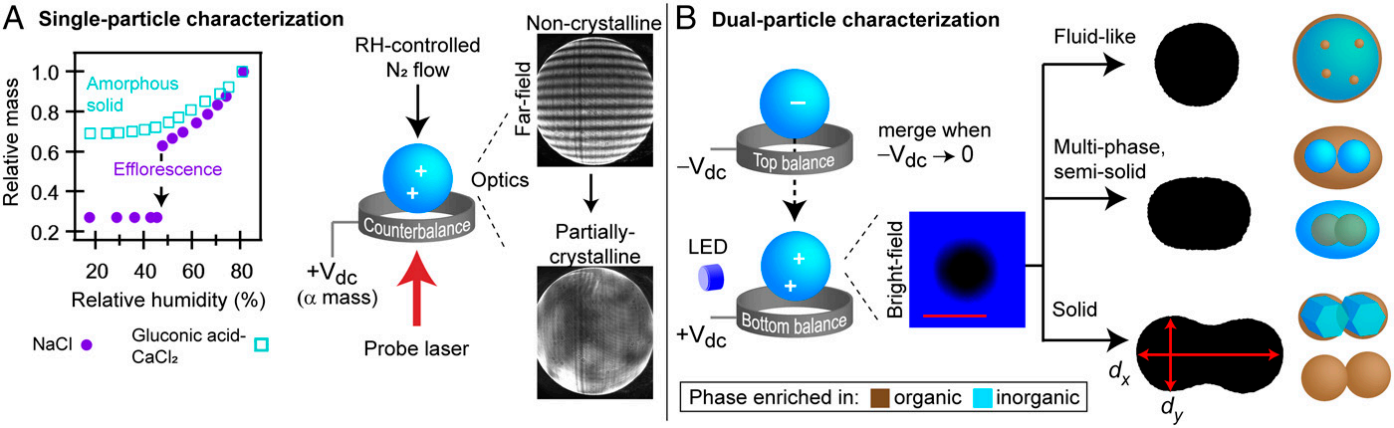
The experimental approach for determining the phase state of levitated aerosol particles. (*A*) Single particles are characterized as a function of RH to determine aerosol phase based on changes in particle mass (*Left Inset*) and particle morphology, as inferred from far-field and bright-field imaging (*Right Inset* and *SI Appendix*, Fig. S2, respectively). (*B*) Dual-particle characterization to identify amorphous phase states, in which two levitated particles are merged together to infer whether the particles are fluid like, semisolid, or solid. When possible, this technique is used to infer the viscosity of merged particles. The morphologies observed in the present study are illustrated.

As shown in Fig. 1, RH-induced phase transformations of aerosols are studied using a dual-balance quadrupole electrodynamic balance (DBQ-EDB) at room temperature (295 ± 2 K) (35). Efflorescence (crystal nucleation and growth with subsequent loss of particle-phase water) can be identified based on mass and morphological changes of singly levitated particles (20 to 30 µm in diameter) exposed to decreasing RH (Fig. 1*A*) (20) (see *SI Appendix*, *SI Methods* for more details). By contrast, amorphous phase changes do not necessarily have any clear morphological or abrupt mass change (22). We thus use our dual-balance technique (35) (Fig. 1*B*) to identify amorphous phase changes associated with the organic fraction of respiratory and growth media aerosols.

An example of detecting efflorescence is shown in Fig. 1*A* for an aqueous NaCl aerosol particle initially levitated at 80% RH. Here, we characterize changes in particle mass relative to 80% RH to be explicit about how experiments were performed; for data in terms of mass growth factors, see *SI Appendix*, Fig. S1. As the RH is reduced stepwise from 80 to 45% (at ∼5% RH intervals), there is a gradual decrease in particle mass as water evaporates from the droplet to maintain an equilibrium water activity. NaCl reaches its saturation concentration at 75% RH but remains as a supersaturated solution because of the kinetic limitations associated with crystal nucleation (36). NaCl efflorescence does not occur until ∼45% RH, which is evident by the abrupt change in particle mass as water is expelled from the particle during crystal growth. Efflorescence can also be identified by changes to particle morphology using far-field laser scatter imaging (Fig. 1 *A*, *Right Inset*). If morphological changes are extensive, bright-field imaging can also be used to detect efflorescence. However, in the case of mixed organic–inorganic droplets, even effloresced particles often remain spherical within the resolution of the imaging system (*SI Appendix*, Fig. S2), and thus, far-field imaging is used intermittently to confirm presumed efflorescence.

In contrast to a phase transition such as efflorescence, amorphous phase changes commonly associated with organic and mixed organic–inorganic aerosols, such as vitrification, gelation, and aggregation, do not have abrupt changes in either particle mass or far-field and bright-field images, as shown in Fig. 1*A*, for an aqueous particle composed of 1:1 (by mole) CaCl_2_:gluconic acid (a composition known to undergo an amorphous phase transition) (22). Thus, to identify potential amorphous changes associated with the organic fraction of respiratory aerosol and growth media, we use our dual-balance technique developed specifically for that purpose (35). As seen in Fig. 1*B*, two oppositely charged particles are simultaneously levitated at a fixed RH and then subsequently merged by removing the voltage applied to the top counterbalance. Bright-field images are binarized, and the aspect ratio of the merged dimer is determined, in which the aspect ratio, d_x_/d_y_, is the ratio of the long-to-short axis. Fluid particles coalesce completely to a spherical shape, in which the timescale of coalescence is proportional to the viscosity of the fluid. Fluid states can be characterized based on viscosity as liquid (<10^2^ Pa·s), semisolid (10^2^ to 10^12^ Pa·s), or solid (>10^12^ Pa·s) (27). In the DBQ-EDB, amorphous particles with a viscosity >10^8^ Pa·s appear completely rigid on the timescale of an experiment (i.e., no discernible coalescence occurs). Many of the compositions studied here were observed to be partially rigid on the timescale of an experiment (i.e., incomplete coalescence occurred). Thus, characterizing each composition in terms of a single viscosity was not possible across all RH, as seen in *SI Appendix*, Fig. S3. In these instances, we characterized phase behavior based on the final aspect ratio of the merged dimers, in which, for semisolid and solid particles, the aspect ratio of the merged particles remains greater than unity, as demonstrated in Fig. 1*B*.

Incomplete coalescence is indicative of a multiphase particle (35). In levitated aerosols, our ability to identify the phase-separated components is limited. Thus, we also used microscopy to examine the morphology of phase-separated aerosols by ejecting merged dimers from the DBQ-EDB onto a microscope slide, as seen in *SI Appendix*, Fig. S4. Peptides and proteins containing the aromatic amino acids (tryptophan, phenylalanine, or tyrosine) fluoresce under ultraviolet (UV) exposure (37–39). Thus, fluorescence microscopy was used to track the location of the organic fraction within aerosols.

With our combination of techniques, we characterize a range of RH-dependent aerosol morphologies relevant to the model respiratory compounds and growth media studied here, as illustrated in Fig. 1*B*.

### Humidity-Dependent Phase Changes of Levitated Aerosol.

**Fig. 2.**
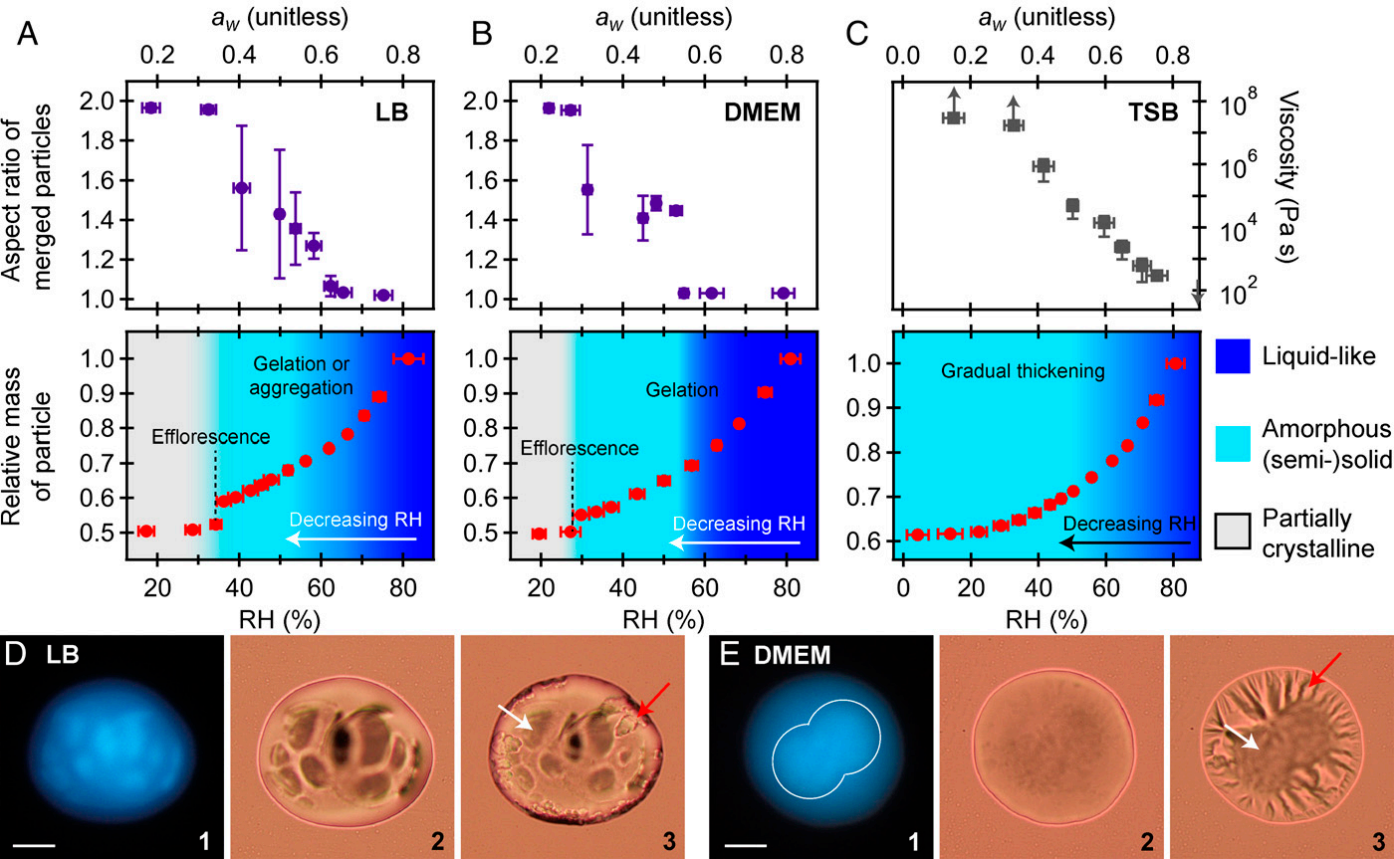
The phase changes of levitated culture growth media (*A*–*C*) and microscopy images of partially coalesced aerosols (*D* and *E*). (*A*–*C*) The top plots are the results from the dual-particle characterization, shown as merged aspect ratio (±1 SD) as a function of RH (±1 SD, with uncertainty propagated from individual RH measurements), except with TSB in which it was possible to infer viscosity for most trials and is thus shown; see the text for estimates of viscosity for other compositions. The lower plots show relative mass as a function of RH for single-particle characterization. Measurements were performed at or near equilibrium, so gas-phase RH was assumed to approximate the particle-phase water activity as *a_w_* = RH/100, as indicated on the plots. For LB and DMEM, phase identification is based on observed changes in the coalescence measurements as well as single-particle mass measurements. TSB did not exhibit any distinct change in phase behavior and remained amorphous at all RH with a gradual increase in viscosity. LB (*D*) and DMEM (*E*) aerosols were merged at 50 ± 2% RH, ejected onto microscope slides, and then imaged in ambient air (∼65% RH). Panel 1 shows fluorescence images. Panels 2 and 3 show brightfield images. In panel 3, efflorescence is induced by blowing dry air over the surface of the slide. For both LB and DMEM, it is seen that incomplete coalescence is due to rigid, organic-dense regions. The organic-dense regions appear in the fluorescence images with higher intensity. For DMEM, the region of highest intensity is outlined in a white line; see *SI Appendix*, Fig. S4*B* for a threshold intensity image. The DMEM particle appears round in panel 2, likely because the surrounding fluid phase has spread on the slide. In both cases, crystallization occurs around the edges of the particle, with the amorphous phase at the center, as indicated by the white (amorphous) and red (crystalline) arrows. (Scale bars, 20 µm.)

**Fig. 3.**
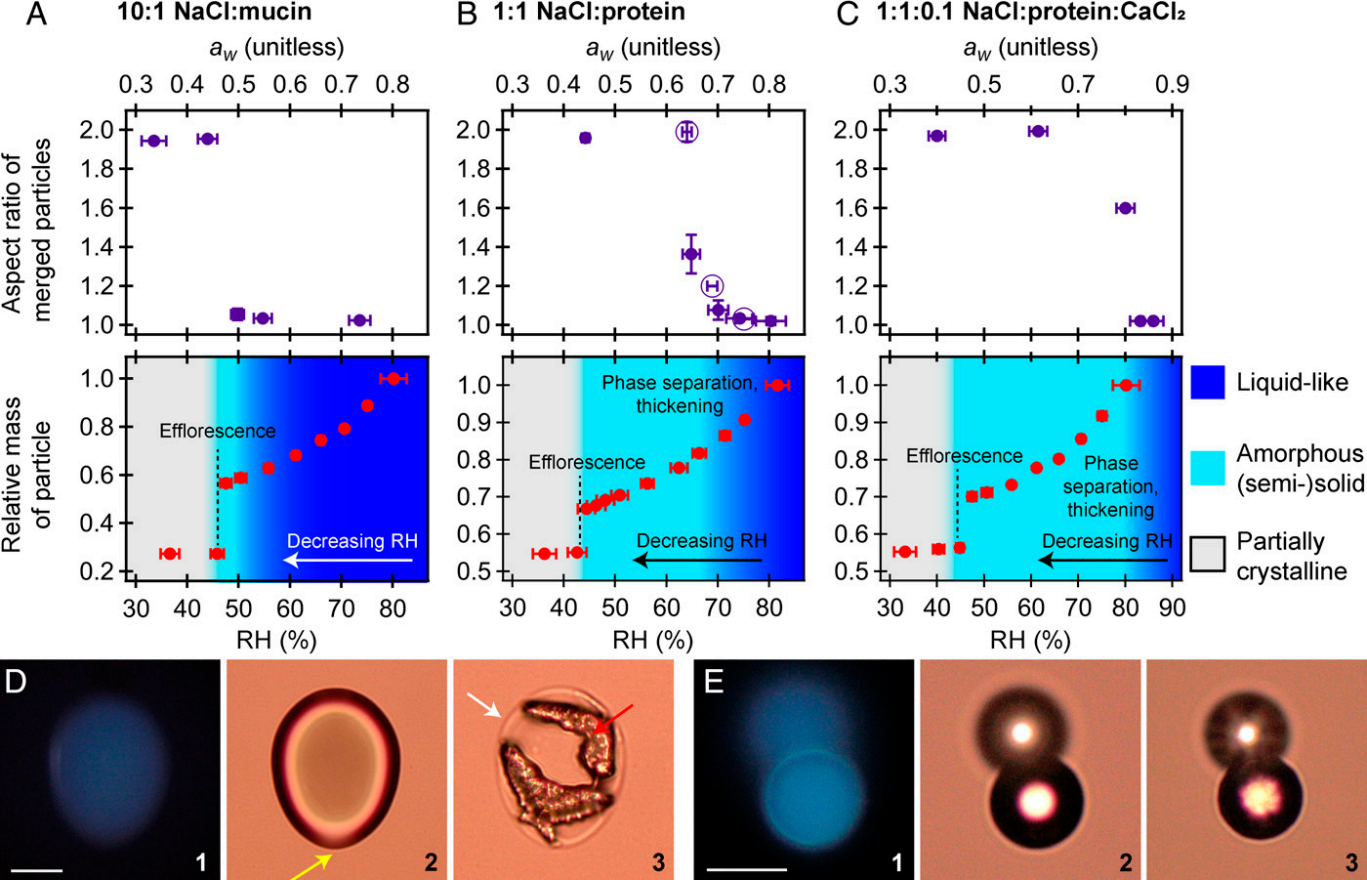
The phase changes of levitated model respiratory compounds (*A*–*C*) and microscopy images of merged aerosols (*D* and *E*). (*A*–*C*) The top plots are the results from the dual-particle characterization, showing merged aspect ratio (±1 SD) as a function of RH (±1 SD, with uncertainty propagated from individual RH measurements). The lower plots show relative mass as a function of RH for single-particle characterization. In *B*, open markers show data points for 1:1 NaCl:albumin. Measurements were performed at or near equilibrium, so gas-phase RH was assumed to approximate the particle-phase water activity as *a_w_* = RH/100, as indicated on the plots. (*D* and *E*) Panel 1 shows fluorescence images. Panels 2 and 3 show brightfield images. In panel 3, efflorescence is induced by blowing dry air over the surface of the slide. Images were collected in ambient laboratory air with an RH of ∼65%. (Scale bars, 20 µm.) (*D*) 1:1 NaCl:protein merged at 68 ± 1% RH. Protein appears uniformly distributed on the particle surface (see *SI Appendix*, Fig. S4*E* for a binary contrast image). Upon efflorescence, there are two distinct NaCl crystals surrounded by an amorphous phase, as indicated with the red and white arrows, respectively. This suggests the protein was phase separated from an aqueous, NaCl-enriched core and that the NaCl cores had not coalesced upon merging. As with DMEM (Fig. 2*E*), the amorphous phases do not appear to have sufficient contrast to be well-resolved in brightfield images prior to efflorescence. However, heterogeneities in the image are visible, as indicated by the yellow arrow in panel 2 and shown in more detail in *SI Appendix*, Fig. S4*F*. (*E*) 1:1 NaCl:albumin merged at 60 ± 1% RH. In the absence of mucin, contact efflorescence was not observed and it was possible to collect a rigid dimer. The dimer did not deposit flat on to the substrate, and one portion was elevated relative to the other. In the images shown, the lower half is in focus. Fluorescence images suggest elevated intensity along the outer edge. Brightfield images show spherical aerosols (within the resolution of the imaging system). Upon efflorescence, the exterior remains spherical with roughening of the interior, as evident by increased refraction of transmitted light. This suggests an amorphous shell around a crystalline core. An additional example is shown in *SI Appendix*, Fig. S4*G*.

Figs. 2 and 3 show the RH-dependent phase behavior for levitated aerosols composed of the growth media and model respiratory compounds, respectively, upon dehumidification.

#### Growth media.

For growth media, DMEM, LB, and TSB were chosen as representative media because they have been used previously in studies of pathogen survival in aerosols and droplets in which a U-shaped RH dependence of virus viability was observed (8, 10). LB (Fig. 2*A*), DMEM (Fig. 2*B*), and TSB (Fig. 2*C*) are mixtures of organic nutrients, such as glucose, and inorganic salts (see *SI Appendix*, Table S1 for the composition of the culture media.) LB and DMEM have similar amounts of NaCl (40 and 48 weight percent [wt.%] by dry mass), while TSB has a much lower-NaCl content (17 wt.%). The organic fraction of LB and TSB is dominated by tryptone (40 wt.%) and peptone (67 wt.%), respectively, while DMEM is dominated by glucose (27 wt.%) and amino acids (12 wt.%). These culture media, thus, represent a range of relevant compositions.

##### LB.

As seen in Fig. 2*A* for LB aerosols, particle coalescence indicates three distinct regions of phase behavior: liquid like, with rapid coalescence and thus nonviscous properties; semisolid, in which viscous, incomplete coalescence was observed; and solid, in which the majority of the particle was nonfluid, following partial efflorescence. At RH above ∼58%, all LB particles rapidly coalesced to a spherical shape with a corresponding viscosity ≤10^2^ Pa·s (the lower limit of viscosity that can be measured in our system), consistent with expectations for liquid aerosols. At ∼60% RH, there is evidence in the laser scatter imaging for aggregates that are small relative to the particle bulk or liquid–liquid phase separation (Movie S1). Thus, the particles are predominately liquid but may have regions of semisolid material. Single-droplet mass measurements show that LB particles respond to decreasing RH with a continuous decrease in mass, further confirming the liquid behavior. Considering this fluid behavior and the fact that the NaCl is supersaturated below ∼75% RH, it is unlikely that virions would have significant protection from deactivation between 60 and 75% RH, consistent with the maximum inactivation of MS2 bacteriophages occurring between 55 to 70% RH in LB (10).

At RH less than ∼58%, merged particles begin to exhibit complex, semisolid behavior. That is, particles do not coalesce completely and are highly viscous. Partial coalescence is evidence for a multiphase system, in which a fraction of the particle remains fluid and a fraction of the particle is rigid (i.e., does not coalesce), as illustrated in Fig. 1*B*. At 50% RH, we estimate the viscosity of the fluid and rigid phase to be ∼10^4^ and >10^8^ Pa·s, respectively, as discussed in *SI Appendix*, Fig. S3*A*. With decreasing RH, the rigid state becomes gradually more pronounced, suggesting a gradual aggregation, growth, or thickening process. There is no abrupt change in single-particle mass measurements between ∼35 to 58% RH, which indicates that efflorescence of NaCl cannot explain this semisolid state. Rather, the semisolid state of LB must be a result of an amorphous phase state. Microscopy images confirm that incomplete coalescence is due to the presence of rigid, organic-enriched, amorphous regions that are surrounded by a viscous fluid, as seen in Fig. 2*D* and *SI Appendix*, Fig. S4*A*. Within the range of RH where a semisolid was observed there was some variability in the coalescence process between different trials, as seen in *SI Appendix*, Fig. S3*A*. This suggests some variability in the size and number of the organic-dense, rigid regions. This likely reflects the stochastic nature of many phase transitions and suggests a nucleation-initiated process (40, 41). However, in all cases, LB existed as an amorphous semisolid.

The exact nature of the rigid phase (e.g., gel or aggregate) is unclear. In addition to NaCl and tryptone, LB contains yeast extract, which is a complex mixture of organic biomolecules and inorganic compounds. Multivalent ions in yeast extract may facilitate a gel state by cross-linking organic molecules (22, 42), and proteins can undergo phase separation through aggregation or liquid–liquid phase separation (28, 43, 44). What is clear is that the aerosols remain noncrystalline in this RH range, as confirmed by far-field laser scatter imaging (*SI Appendix*, Fig. S2) and microscopy (Fig. 2*D*). Regardless of the molecular-level structure of the semisolid, viscous and gel states may both have a protective effect on the survival of viruses between 35 and 58% RH by inhibiting the diffusion of reactants (27).

At ∼35% RH, both particle coalescence and single-particle mass measurements indicate a transition to a solid state. Merged particles remain rigid, and there is an abrupt change in single-particle mass, consistent with efflorescence of the NaCl. With repeat trials, the efflorescence RH, RH_eff_, was 35 ± 3% RH (*n* = 5). This RH_eff_ can be interpreted as the RH at which the efflorescence probability, *P*_eff_, is 1.0 ± 0.4 under these experimental conditions (see *Materials and Methods*, *Statistical Analysis*). Efflorescence of LB occurs at a lower RH than aqueous NaCl (∼45% RH), likely due to the presence of organic compounds that hinder nucleation (23–25) (see *SI Appendix*, *Factors Influencing Phase Changes* for additional discussion). Efflorescence of the inorganic fraction, along with further thickening of the amorphous phase through dehydration, will likely offer maximum protection of pathogens by hindering diffusion and thus reactivity.

##### DMEM.

As seen in Fig. 2*B*, DMEM aerosol particles also exhibited three distinct phases. Using the same characterization process as for LB, DMEM is observed to be in a liquid state above ∼53% RH. At ∼53% RH, coalesced droplets exhibit a compressed, partially rigid structure, in which the average aspect ratio was ∼1.4 (see Fig. 1*B* for an example). Merged dimers adopted a compressed shape rapidly (<3 s; *SI Appendix*, Fig. S3*B*), so we conclude particles must be phase separated, comprised of a relatively nonviscous fluid and a rigid semisolid. As seen in Fig. 2*E*, microscopy images confirm this morphology. Incomplete coalescence at long timescales suggests that the rigid phase has an equivalent viscosity of >10^8^ Pa·s. The abrupt change in morphology over a narrow RH range is consistent with a gel transition (22, 35). DMEM contains low-mass, carboxylated organic compounds (e.g., the amino acids) in addition to divalent ions, including Ca^2+^. A gel transition involving carboxylated organics and Ca^2+^ has previously been shown to occur at ∼50% RH in levitated aerosols. In DMEM, it is likely that the divalent ions are cross-linking the amino acids to form a gel state (22). A solid state is observed when efflorescence occurs at 31 ± 3% RH (*P*_eff_ = 1.0 ± 0.4, *n* = 5). Similar to LB aerosols, efflorescence of NaCl is hindered relative to aqueous NaCl alone.

##### TSB.

As seen in Fig. 2*C*, TSB aerosols gradually transition from a fluid state to an ultraviscous, amorphous solid state, as evident in the dual-particle coalescence measurements. For TSB, we report viscosity rather than aspect ratio because complete coalescence was consistently observed, as seen in *SI Appendix*, Fig. S4*C*, except at high viscosity and low RH. Aerosols with a viscosity >10^2^ Pa·s are typically described as semisolid because diffusive limitations can influence observations that occur on the timescales of a typical experiment (seconds or longer) (23, 27, 45). It is thus likely that TSB exhibits semisolid behavior below ∼70 to 80% RH, in which the viscosity is ∼10^2^ to 10^3^ Pa·s. However, there is no distinct transition, and the transition to semisolid behavior occurs across a range of viscosity. As shown in *SI Appendix*, Fig. S5, viscosity increases rapidly following initial trapping and is >10^4^ Pa·s at 50% RH after ∼1 min. Below ∼40% RH, TSB behaved as an amorphous solid, and the high viscosity made complete coalescence impractical to observe, as seen in *SI Appendix*, Fig. S4*D*. The viscosity reported is thus a lower limit, based on observation time (22). The gradual increase in viscosity as RH is decreased is consistent with expectations for an ultraviscous or glass-forming fluid (35). As seen in the single-particle mass measurements, there is no clear efflorescence transition with TSB, as confirmed with microscopy (*SI Appendix*, Fig. S4*C*). Because of the high viscosity and high-organic content, efflorescence of NaCl is likely completely hindered in aerosols of TSB.

#### Model respiratory compounds.

For model respiratory compounds, we focused on mixtures of salt and protein. The composition of biological respiratory fluid can vary across individuals and depend on state of illness and origin in the respiratory tract (18, 46–48), and it is not practical to study every possible respiratory fluid composition. We thus focus here on understanding the fundamental processes that are occurring with mixtures composed of the dominant components of respiratory fluid (NaCl and protein) and CaCl_2_, the latter of which is known to cross-link proteins and other molecules into a gel state (22, 42). For NaCl–protein mixtures, we studied the effect of varying protein content using a low-protein mixture (10:1 NaCl:mucin by mass; Fig. 3*A*) and a high-protein mixture (1:1 NaCl:protein, with a protein content of 9:1 albumin:mucin by mass; Fig. 3*B*). For comparison, 1:1 NaCl:albumin was also studied. The high-protein mixture was also studied with CaCl_2_ at 10:1 NaCl:CaCl_2_ by mass (Fig. 3*C*). Although real saliva and lung fluid are composed of more compounds than salt and protein, these compounds comprise a significant portion of the inorganic and organic fraction (46–49), and our observations can thus provide fundamental insight into the morphology and phase transitions of expelled respiratory particles.

##### Low-protein content.

With the low-protein composition (Fig. 3*A*), the phase behavior of levitated aerosol is similar to that of aqueous NaCl. At all RH >45%, aerosol particles were predominately liquid, although far-field imaging (Movie S1) suggests the presence of small protein aggregates or emulsions that are too small to prevent or inhibit coalescence. Subtle, semisolid behavior is not observed until <50% RH, in which the average aspect ratio of merged dimers is just above unity (1.06 ± 0.03), so any semisolid portion is small relative to the bulk of the droplet. Phase separation (referred to here as the formation of any protein-enriched phase through, for example, aggregation or liquid–liquid phase separation) is possible in protein solutions and is likely responsible for any semisolid behavior (44, 50–52). The transition to a solid particle occurred at the characteristic efflorescence RH of NaCl at 45 ± 2% RH (*P*_eff_ = 1.0 ± 0.4, *n* = 5). These results indicate that the phase behavior of respiratory particles with a high mass fraction of NaCl will be similar to the characteristic efflorescence/deliquescence behavior of NaCl, consistent with previous observations (53).

##### High-protein content.

With the high-protein mixture, the RH region over which semisolid behavior was observed was significantly expanded (relative to the low-protein mixture), as seen in Fig. 3*B*. Merged dimers began exhibiting semisolid behavior at ∼70 to 75% RH, in which viscous flow was observed, as shown in *SI Appendix*, Fig. S3*D*. Above 75% RH, coalescence was rapid, and viscosity was <10^3^ Pa·s. At 70% RH, the inferred viscosity was ∼10^5^ Pa·s (*SI Appendix*, Fig. S3*D*). Below ∼65% RH, the aspect ratio of merged dimers could not be determined clearly because contact efflorescence was observed (*SI Appendix*, Fig. S6). That is, upon the merging of two particles, both particles were observed to effloresce. Contact efflorescence of NaCl has been described previously (21, 36, 54). Aerosols of 1:1 NaCl:albumin (i.e., without mucin) exhibited similar phase behavior as the mixture with mucin, but no contact efflorescence was observed. As seen in Fig. 3*B*, NaCl-albumin aerosols were rigid at RH < 65%, suggesting an ultraviscous state (>10^8^ Pa·s). We note that we saw no evidence of heterogeneous efflorescence in the single-particle studies, with efflorescence at the homogeneous efflorescence RH for NaCl of 44 ± 3% (*P*_eff_ = 1.0 ± 0.4, *n* = 5), as evident in the single-particle measurements.

It is notable that NaCl efflorescence was observed despite the highly viscous state (demonstrated in *SI Appendix*, Fig. S3*D*) and the high organic fraction. This suggests that phase separation has occurred, in which the protein has phase separated from the aqueous inorganic (29, 50). A viscous, protein-enriched shell with a nonviscous, aqueous inorganic core would explain how we can observe an ultraviscous, semisolid phase in the coalescence measurements but also observe rapid and uninhibited NaCl efflorescence. Evidence for this morphology is seen in Fig. 3 *D* and *E*. Furthermore, as shown in *SI Appendix*, Fig. S7, Mie resonance measurements of single particles supports the notion that phase separation is occurring in NaCl–protein mixtures (*SI Appendix*, *SI Methods*). Mie resonance spectra (55) indicate a reversible loss of spherical morphology (i.e., heterogeneities at the aerosol surface) at <70% RH. Efflorescence occurred at ∼45%, consistent with coalescence data.

##### High-protein content with CaCl_2_.

In addition to the simple NaCl–protein mixtures, we moved toward more complex systems by including CaCl_2_ in the high-protein mixture at a ratio of 1:1:0.1 NaCl:protein:CaCl_2_ by weight. As seen in Fig. 3*C*, the presence of Ca^2+^ broadens the range over which levitated aerosol particles are (semi)solid (relative to the other salt–protein mixtures), with a semisolid state at ∼80 to 85% RH. In this mixture, the Ca^2+^ is likely cross-linking the protein to create a gelatinous matrix (22, 42).

Although coalescence measurements indicate an amorphous, (semi)solid phase as high as 80 to 85% RH with the NaCl–protein–CaCl_2_ mixtures, this did not inhibit the efflorescence of NaCl, which occurred at 45 ± 2% RH (*P*_eff_ = 1.0 ± 0.4, *n* = 5). As demonstrated in *SI Appendix*, Fig. S8, merged NaCl–protein–CaCl_2_ particles are rigid at intermediate RH, but these rigid dimers still effloresce at ∼45% RH. Similar to the 1:1 NaCl:protein mixture, this points to a phase-separated morphology, potentially in which a gelatinous shell, which is sufficiently rigid so as to prevent coalescence, is covering an aqueous inorganic core, as suggested by Vejerano and Marr with model respiratory compounds (18). Microscopy evidence for this morphology is shown in *SI Appendix*, Fig. S4 *H* and *I*.

In general, our results demonstrate that elevated protein content increases the RH range over which respiratory aerosols are likely to exist in a (semi)solid state. Divalent ions will also enable a wider range of semisolid phases than protein and NaCl alone. Although our model systems do not contain the full chemical complexity of respiratory fluid, it is clear from these results that considering NaCl efflorescence alone is inadequate to describe the phase state of respiratory emissions as well as aerosolized culture growth media.

### Phase Changes of Droplets Deposited on a Surface.

**Fig. 4.**
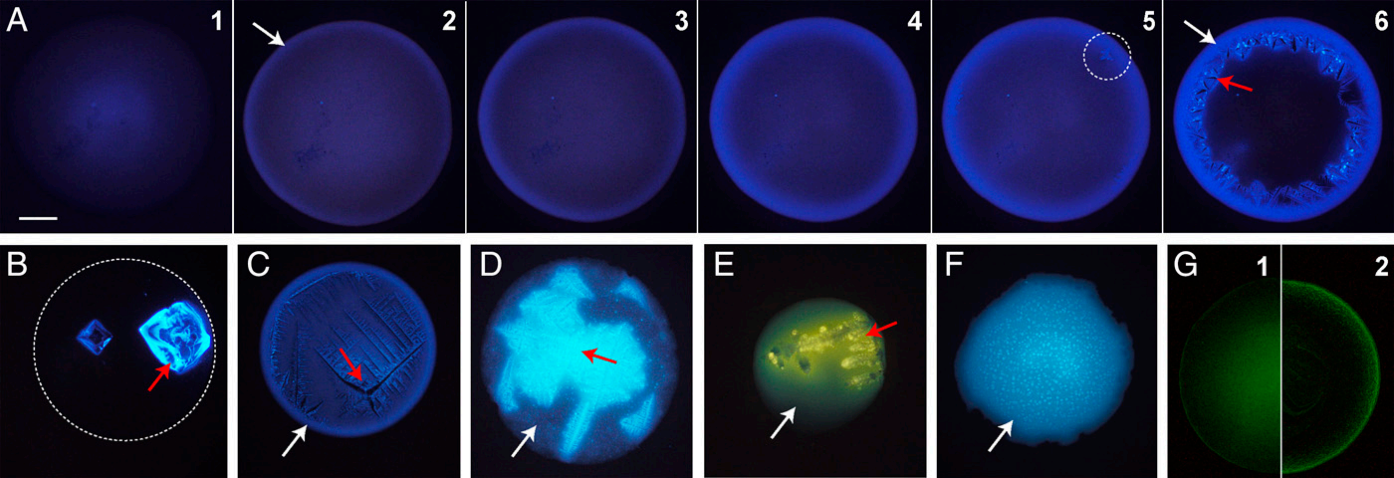
Fluorescence microscopy images of evaporating droplets deposited on a hydrophobic glass surface at ∼40% RH. (*A*) 1:1 NaCl:protein, with images shown as a function of time at *t* = 0 (frame 1), 13 (frame 2), 16 (frame 3), 19 (frame 4), 21 (frame 5), and 26 min (frame 6). Images *B*–*F* show the morphology of deposited droplets after 30 min of evaporation. (*B*) 10:1 NaCl:mucin. The white dashed line marks the outline of the droplet (*SI Appendix*, Fig. S9). (*C*) 1:1:0.1 NaCl:protein:CaCl_2_. LB (*D*), DMEM (*E*), and TSB (*F*). (*G*) The distribution of 100-nm diameter fluorescent nanobeads in 1:1 NaCl:protein after initial deposition (frame 1) and after 30 min of evaporation (frame 2). (Scale bar, 200 µm.) White arrows indicate amorphous phases, and red arrows indicate crystalline phases. Contact angles are provided in *SI Appendix*, Table S2.

To complement the studies of levitated aerosol and to explore the phase changes of these systems deposited on a surface, we collected microscopy images of evaporating growth media and model respiratory droplets deposited on hydrophobic (Fig. 4 and *SI Appendix*, Fig. S9) and hydrophilic (*SI Appendix*, Fig. S10) glass slides. Droplets (3 µL initial volume) were exposed to ambient laboratory air (30 to 45% RH) while monitoring the evaporation process.

Fluorescence microscopy images of droplets on hydrophobic slides are shown in Fig. 4 (corresponding bright-field images are shown in *SI Appendix*, Fig. S9). Fig. 4*A* shows the time-dependent evaporation of 1:1 NaCl:protein droplets exposed to 40% RH laboratory air. Immediately, after droplet deposition (*t* = 0 min), the protein fraction, which appears blue in the image, is distributed across the droplet with the highest intensity at the center (where the droplet is thickest; see *SI Appendix*, Fig. S11 for an intensity line profile). As the droplet evaporates, a protein-enriched shell forms, evident by an increase in fluorescence along the outer edge, as seen at *t* = 13 min. This is consistent with the evidence for phase separation observed in aerosols, with a protein-enriched shell and an aqueous core that is dominated by NaCl (29, 44). Although the exact mechanism of phase separation is unclear from these images, Mie resonance measurements on aerosols (*SI Appendix*, Fig. S7) suggest that phase separation occurs through the formation of aggregates or other protein-dense regions that accumulate at the interface. In the case of evaporating droplets, Marangoni flow may facilitate the transport of protein-dense regions to the interface (i.e., the coffee ring effect).

At *t* = 21 min, NaCl crystal nucleation and growth is evident, with nucleation appearing to originate at the organic–inorganic interface. We note that the growing crystalline phase appears to have a higher fluorescence intensity than the surrounding solution. NaCl crystals are nonfluorescent (confirmed in *SI Appendix*, Fig. S10*J*). Thus, this increased intensity is likely due to scattering and reflection of the excitation and emission wavelengths off of the irregular facets of the growing crystal, which leads to an enhancement in the observed fluorescence. As seen at *t* = 26 min, after crystal growth is complete, there is no visible presence of crystals along the outer edges of the droplet. Rather, the NaCl crystals grow along the inner edge of the amorphous, protein-enriched phase. As shown in *SI Appendix*, Fig. S12, this morphology is also observed in acoustically levitated microliter droplets in the absence of a surface.

Of the remaining compositions, all evaporated in 30 min or less and had clear evidence of an amorphous phase state, except the low-protein mixture (Fig. 4*B*), consistent with the aerosol levitation experiments. No clear protein shell is apparent in the low-protein droplets in Fig. 4*B*, potentially because of adsorption of a thin layer of protein to the hydrophobic surface (56). However, higher-magnification images suggest phase separation has still occurred, as seen in *SI Appendix*, Fig. S9*G*. We note that, in Fig. 4*B*, the high fluorescence intensity associated with the NaCl crystals is likely due to enhanced fluorescence due to scattering and reflection and is not indicative of a high protein content in the NaCl crystal. As seen in Fig. 4*C*, the presence of Ca^2+^ leads to a different crystal habit than with NaCl–protein alone (Fig. 4*A*), although there remains a clear protein-enriched shell.

LB (Fig. 4*D*) and DMEM (Fig. 4*E*) also exhibited phase-separated morphology, with an amorphous and crystalline phase. Rather than a distinct organic shell, the amorphous phase was nonuniformly distributed throughout the droplet. LB also transitioned to a phase-separated morphology in the presence of *Escherichia coli*, as seen in *SI Appendix*, Fig. S13, suggesting that our observations of a semisolid state will not be significantly changed by propagating pathogens, although hygroscopicity may be changed to some extent, as indicated by Fernandez et al. (57).

In contrast to the other compositions, TSB droplets (Fig. 4*F*) did not have clear phase separation; the organic content was distributed throughout the droplet, with no apparent crystalline phase. On hydrophilic surfaces (*SI Appendix*, Fig. S10), TSB, LB, and low-protein droplets exhibited slightly different morphologies. However, the trends remained the same, with an amorphous phase state observed in all cases.

Although the final evaporated morphology and crystal habit varied somewhat between different droplet compositions, an amorphous phase state was the unifying characteristic. Particularly notable was the phase separation observed for compositions with high-protein content and salts because these components dominate respiratory fluid. Considering the general protective effect of proteins on viral survival, the presence of a protein-enriched phase has implications for the surface and airborne survival of pathogens.

To qualitatively explore whether pathogens may be influenced by the protein-enriched shell of NaCl–protein solutions, we doped the 1:1 NaCl:protein solution with amine-functionalized 100-nm fluorescent polystyrene latex spheres (PSLs). SARS-CoV-2 is ∼100 nm in diameter (58). Thus, the PSLs used here will have similar diffusive mobility. As seen in Fig. 4*G*, the PSLs are initially distributed uniformly through the droplet (Fig. 4*G*, frame 1) and then partition to and become concentrated near the droplet interface (Fig. 4*G*, frame 2). The location of the PSLs in the evaporated droplet is within the region that is enriched in protein. As seen in *SI Appendix*, Fig. S14, the PSLs are indeed embedded in the viscous protein phase, and few exist entirely at the air–fluid interface. The presence of proteins may explain why fluorescent particles did not partition to the air–droplet interface of smaller droplets composed of model respiratory compounds (18) and saliva (13). Considering viruses to be organic colloids, it would be reasonable to expect them to partition to an interface (the extent of partitioning being dependent on virus surface composition and size) (59), in which inactivation can occur (60). However, as shown here, proteins can become enriched at the surface, which can inhibit surface partitioning.

## Discussion

### The Potential Role of Aerosol and Droplet Phase on the RH-Dependent Survival of Pathogens.

Laboratory observations have suggested that efflorescence of NaCl can have a protective effect on viral stability and can help explain the U-shaped RH dependence of virion survival (14). Focusing on NaCl efflorescence alone, however, cannot account for the protective effect of proteins (3) and extracellular material (2) nor for the U-shaped survival of viruses in aerosol compositions with low salt content that were not observed to effloresce (e.g., TSB) (10). Based on our observations of organic- and protein-enriched, amorphous phase states in model respiratory compounds and growth media, we propose here an additional factor influencing the survival and, thus, transmission of pathogens: hindered diffusion within amorphous (semi)solid states.

**Fig. 5.**
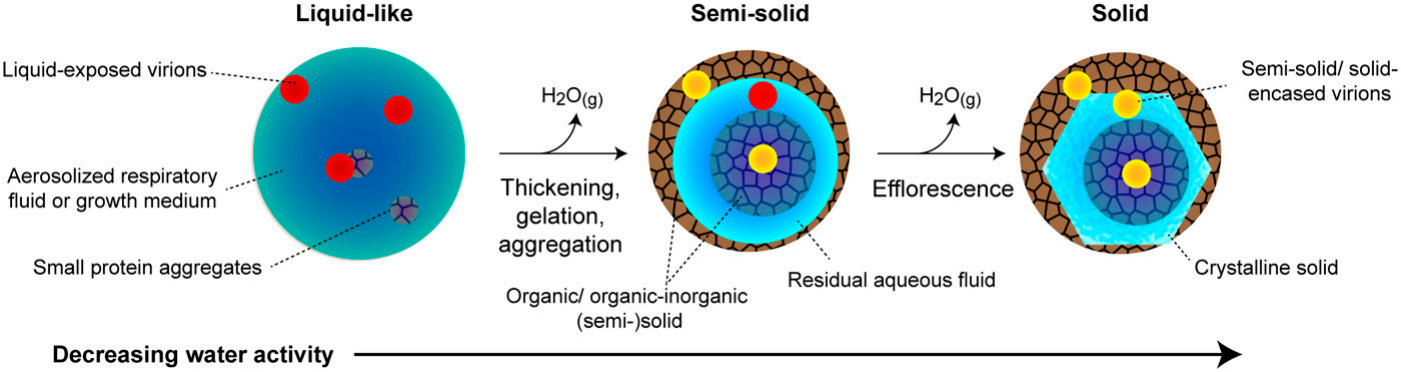
The different microenvironment pathogens can be exposed to in aerosols and droplets composed of growth media and respiratory compounds. With decreasing RH, amorphous, (semi-)solid phases can form at intermediate to low *a_w_*, potentially shielding pathogens from inactivation by inhibiting diffusion of reactants.

As illustrated in Fig. 5, if an aerosol or droplet contains pathogenic material, each phase state would present a different microenvironment with distinct physicochemical properties. At high RH, the liquid-like state of aerosols and droplets will leave virions exposed, although solute concentrations will be lower, and disinfection may proceed at a slower rate. As RH is decreased, solute concentrations increase, which is expected to increase the rate of inactivation (8, 10, 15). We posit that, with decreasing RH, this increased rate of inactivation due to increasing solute concentration will be partially offset by the formation of phase-separated organic and organic–inorganic amorphous phases that hinder diffusion of reactants.

Organic and organic–inorganic (semi)solid states, such as glassy, ultraviscous, and gelatinous phases, can exhibit properties that are similar to a solid on timescales relevant to pathogen survival (hours to days). For example, diffusion rates of molecules through a gelatinous or glassy matrix can be substantially hindered, with potential diffusional mixing timescales of hours to years for a (semi)solid 1-µm diameter aerosol (27, 61) (*SI Appendix*, *Effects of Diffusive Limitations*). Viscous states thus tend to slow chemical reactions, in which reaction rates are directly proportional to the diffusion coefficient of reacting solutes and inversely proportional to the viscosity of the reaction medium (62, 63). Any biological material, including pathogens, encased in a gelatinous or glassy matrix may thus be protected from oxidative damage or solute effects. Indeed, this protective effect is the basis for utilizing polymeric gels and glasses as a means for microencapsulation and preservation (64, 65). The organic fraction of respiratory aerosol and growth media thus represent a phase in which bioactive material can be protected. The effect will become more pronounced as RH is reduced further, increasing viscosity and further hindering diffusion (27, 61). Upon efflorescence (if it occurs), aerosols and droplets transition to a partially crystalline state, which likely provides further protection from inactivation (14).

**Fig. 6.**
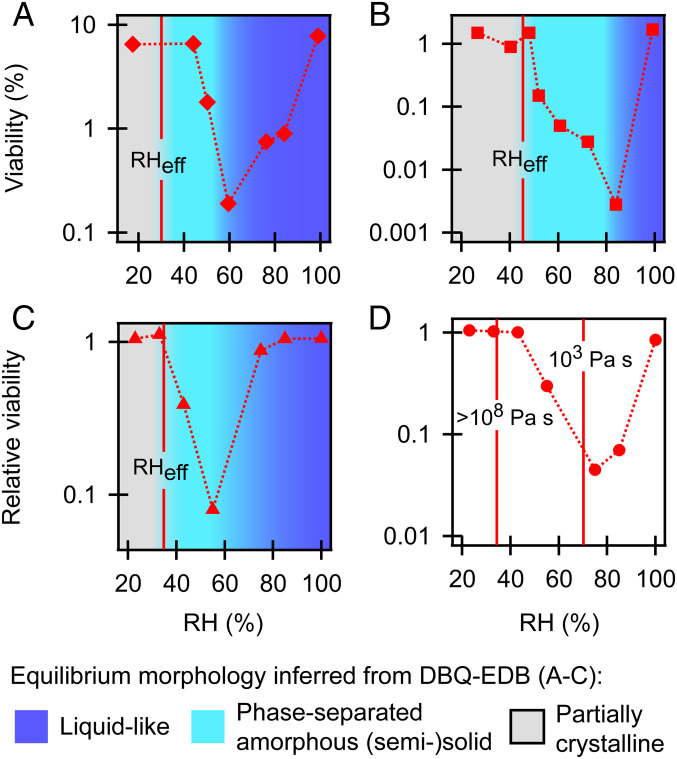
Plots of previously reported virus viability data in a range of compositions compared to our observations of inferred equilibrium phase states from Figs. 2 and 3 (i.e., when *a_w_* ≈ RH/100). RH_eff_ indicates our observed efflorescence transition for the medium used. (*A*) Influenza in DMEM droplets from Yang et al. (8). (*B*) Influenza in mucus droplets from Yang et al. (8). We use our observations of NaCl-protein-CaCl_2_ aerosols as proxies for mucus. (*C*) MS2 in LB aerosols from Lin and Marr (10). (*D*) Phi-6 in TSB aerosols from Lin and Marr (10). No RH_eff_ is indicated since TSB was not observed to effloresce in the present study. Since no distinct transition in phase behavior was observed for TSB, we indicate the RH values at which the viscosity of TSB was 10^3^ and >10^8^ Pa·s (respectively, the approximate lower and upper limit of viscosity observable in our system). The RH of minimum viability of phi-6 in TSB is coincident with an equilibrium viscosity of ∼10^3^ Pa·s. This correlation may be because, at ∼10^3^ Pa·s and greater, diffusive mixing timescales of solutes in micrometer-sized aerosols, as used by Lin and Marr (10), can be comparable to or longer than the timescale of the viability experiments (minutes to hours) (45) (*SI Appendix*, *Effects of Diffusive Limitations*), which may help explain the recovery of phi-6 in TSB aerosols at <75% RH. At >10^8^ Pa·s, TSB aerosols behaved as amorphous solids on the timescale of the coalescence measurement (hours). Semisolid behavior is thus expected within this range of viscosities. In all cases (*A*–*D*), the recovery in virus viability is coincident with RH values at which we observe an amorphous, semisolid state, demonstrating that salt efflorescence alone is inadequate to explain the RH-dependent viability of viruses. These RH values and viscosities should not be taken as definitive thresholds for the recovery in viral viability because of additional factors that influence viral survival, as well as the existence of time-dependent spatial gradients in phase and viscosity in which RH and *a_w_* are not necessarily equivalent. See text for additional discussion.

To explore whether an amorphous, semisolid state may indeed be influencing viral survival, we compared our observations to previously reported datasets of viral survival from Yang et al. (8) and Lin and Marr (10). These studies were chosen for comparison because there are a sufficient number of RH values for a relevant comparison to our data and because the timescale of evaporation was short relative to the total observation time. In Yang et al. (8), droplets evaporated in ∼30 min, which is short, relative to the observation time of 3 h for DMEM and 2 h for mucus. In Lin and Marr (10), LB and TSB aerosols likely evaporated in seconds or less, which is short, relative to the 1-h observation time. As seen in Fig. 6, detailed knowledge of the aerosol phase state, including semisolid phases, can help explain the RH-dependent viability of viruses in a range of aerosol and droplet compositions. The RH at which a semisolid phase is observed in our studies is coincident with recovery of virus viability in previous studies. As RH is reduced further, the extent of semisolid behavior and viscous properties gradually increases, which corresponds to a gradual recovery in virus viability.

The existence of an amorphous phase state that influences pathogen survival has been suggested previously (10, 16, 18). Here, our observations provide empirical evidence to support that supposition and strongly suggest that there are four equilibrium RH regions that dictate virus viability: 1) physiological conditions (high RH, ∼99 to 100%), in which solute concentration is low and harmless to pathogens; 2) concentrated conditions, in which elevated solute concentrations are high, and particles are in a liquid-like state (and diffusion is not hindered) (∼75% RH, depending on composition); 3) semisolid conditions, in which increasing viscosity (and decreasing diffusion) offsets increasing solute concentrations (∼45 to 75% RH, depending on composition); and 4) dry conditions, in which a substantial fraction of water is lost, and the formation of a crystalline or amorphous solid inhibits inactivation (<45% RH). This conceptual framework predicts that increased organic fraction of aerosols and droplets will, in general, shift the RH of minimum viability to higher RH and increase overall viability. This is consistent with observations. For example, proteins typically have a protective effect on viral survival (3). Furthermore, the organic fraction of human saliva can explain why Fedorenko et al. (13) observed enhanced survival and a higher RH of minimum viability for bacteriophages in saliva droplets relative to SM buffer (which is dominated by NaCl and other salts). Similarly, relative to a salt solution, Zuo et al. (66) observed enhanced survival of MS2 in aerosols doped with mucin and composed of TSB (75% organic by dry weight), further suggesting that amorphous, semisolid-phase states are influencing viral survival.

Although this description of semisolid-phase states can qualitatively account for trends in viral survival, we emphasize that a comprehensive description of aerosol and droplet composition must consider additional factors. Notably, RH is only an appropriate indicator of particle-phase water content and, thus, solute concentration and phase state, under equilibrium conditions. In aerosols, quasiequilibrium with RH is reached relatively quickly [i.e., a majority of the water will evaporate in seconds or less (33) and a semisolid-phase state can form in <1 min, as demonstrated in *SI Appendix*, Fig. S5]. These timescales decrease with decreasing aerosol diameter and are short, relative to the timescale of survival for most viruses. However, with increasing viscosity and particle diameter, spatial gradients become increasingly significant, and the timescale to reach equilibrium increases. For example, evaporation timescales can be significant in droplets (minutes to hours). Thus, in addition to the RH regions stated here, this evaporation period is an important consideration for understanding viral survival in droplets (34). We note that even under quasiequilibrium conditions, spatial heterogeneities due to phase separation or viscosity gradients suggest that virions in the same particle can be exposed to drastically different microenvironments, depending on location. Furthermore, because of the stochastic nature of some phase transitions, as suggested with observations of LB (*SI Appendix*, Figs. S3*A* and S5*B*), there may be particle-to-particle variability in pathogen viability. Because of this variability, it is likely that viral viability reflects a statistical average of the outcomes in these different microenvironments. Indeed, spatial heterogeneities and particle-to-particle variability may explain why there is not a complete recovery in viral viability at intermediate RH in the studies shown in Fig. 6.

We note that virus inactivation will be influenced by many complex factors beyond the media phase state. These factors can include surface structure, specific solute effects, location in the particle (e.g., surface or bulk), and sunlight exposure, among other factors (1, 3, 9, 60). Many of these factors are likely interdependent, with a complex interplay between pathogen surface structure, solvent (water), and solutes. Considering virions to be large solutes or colloids, certain virions may incorporate into gel structures or serve as protein aggregation sites, while others may preferentially remain in the aqueous phase. Indeed, different viruses can exhibit different, RH-dependent behavior, even in the same media. However, solute effects are all slowed in a viscous or (semi)solid state because of diffusive limitations (27, 61).

Viruses with rapid disinfection kinetics or highly unstable surface structure may be less protected by a semisolid state. For example, Harper et al. (67) observed a U-shaped viability curve for poliomyelitis in aerosols after 5 min of aging, but the recovery in viability at low RH was small compared to the viability at high RH. For this virus, this may reflect rapid inactivation during the evaporation phase. In particular, extremely rapid inactivation during evaporation may evade the protective effect of an amorphous state if inactivation occurs on faster timescales than the formation of the semisolid phase (seconds to minutes). Thus, because of variations in viral structure and mechanisms of inactivation, which are not entirely known, the RH at which viral viability is preserved may vary to some extent, particularly in media that do not exhibit a distinct phase separation (e.g., TSB). However, gel transitions and phase separations often occur within a relatively narrow equilibrium RH (*a_w_*) range (i.e., a critical solute concentration) and can lead to abrupt discontinuities in physical properties (22, 29, 44, 68). Such phase transitions, as observed with, for example, DMEM and LB, may have an effect on viral viability at the transition point, depending on the location of the pathogen in the particle, the mechanism of inactivation, and rate of disinfection relative to the formation of the amorphous phase. This highlights the importance of further understanding the spatial and temporal evolution of aerosols and droplets during evaporation, the location of virions in suspension media, and the time dependence of pathogen viability (69). Overall, even in the absence of a phase transition, our observations suggest that increasing the protein content of the medium is likely to increase viability, consistent with observations (8).

Although the mechanism of viral inactivation is not known, hindered diffusion is expected to impact any solute-induced mechanism of disinfection. For example, long mixing timescales in an amorphous (semi)solid are likely to provide protection against gas-phase disinfectants, such as ozone, which must partition to the surface of the particle and then diffuse into the bulk (27). While diffusive mixing timescales are dependent on compartment size, the rates of chemical reactions are slowed in a viscous state independent of length scales (62, 63). We thus expect a semisolid state to also provide protection against solutes in solution, such as salts. Thus, the protective effects of amorphous organic phase states are likely general, with implications for understanding disease transmission.

### Implications for the Transmission of Pathogens under Ambient Conditions.

There are several implications for the presence of an organic-based, protective phase of respiratory aerosols and droplets. For example, this suggests that pathogen survival may not only depend on temperature and RH but also where the associated respiratory particle originated in the respiratory tract. Respiratory fluid composition varies along the tract (70), and sites with higher organic content may produce respiratory particles containing pathogens that are better equipped to survive in the environment because of their surrounding matrix. Some researchers hypothesize that respiratory particles containing viable influenza A virus can originate from deep in the lungs (5, 71), where total organic concentrations are thought to be higher than in saliva and other areas of the respiratory tract (70). Furthermore, it was recently reported that cough-generated aerosols from healthy subjects are primarily organic (∼90% organic by volume of nonvolatile components) (72). With such a high organic fraction, it is highly likely that cough aerosols exist in a semisolid state that can protect pathogens from inactivation, even at intermediate RH (50 to 70% RH). By contrast, large respiratory droplets are thought to originate primarily from the oral region where saliva is present (73). An additional consideration is that illness can increase the production of mucus in infected hosts. Thus, pathogen viability likely depends on the extent of illness.

### Concluding Remarks.

The transmission of pathogens from host to host is a complex process involving many factors. Respiratory aerosol and droplet phase state is one potential factor that can influence the viability of airborne and surface-deposited, infectious material. In this study, we illuminated important aspects of aerosol and droplet evaporation and associated phase changes, demonstrating that there exist amorphous phase states that are likely influencing the survival, and thus transmission, of pathogens carried by respiratory emissions.

Our observations also indicate that for laboratory studies of pathogen survival in aerosols and droplets, particle size, media composition, and supporting substrate (if any) should be carefully considered when making extrapolations to ambient conditions. This is because all of these factors can influence phase changes and the existence of spatial heterogeneities in phase and viscosity (see *SI Appendix*, *Factors Influencing Phase Changes* for additional discussion). If a growth medium is used as the aerosol/droplet suspension, it would be prudent to select a medium that exhibits comparable phase behavior to that of various respiratory fluids to ensure laboratory observations are translatable to human respiratory emissions originating in different locations in the respiratory tract. Growth media, such as DMEM, which do not contain a significant fraction of protein, may be more comparable to respiratory fluid with the addition of albumin or mucin.

The presence of amorphous phase states may contribute to the RH and temperature-dependent survival of extracellular pathogens. Low RH and temperature favor the formation of amorphous solids, consistent with the observations that virions survive best at low RH and low temperature (although many factors contribute to temperature-dependent survival; see *SI Appendix*, *Discussion of Temperature Effects* for further discussion). The slow diffusion of disinfectants in amorphous solids will effectively microencapsulate pathogens in respiratory particles. Respiratory particles with higher-protein content may thus protect virions from disinfection at a higher RH than would respiratory particles with a higher-inorganic content. These observations will help interpret laboratory studies as well as inform mitigation strategies for controlling the spread of pathogens.

## Materials and Methods

### Aerosol Levitation.

Aerosol particles, 20 to 30 µm in diameter, were levitated in a DBQ-EDB, as described extensively elsewhere (35). The DBQ-EDB was used for both single-particle and dual-particle characterization, as described in greater detail in *SI Appendix*, *SI Methods*. In brief, aerosol particles are generated from aqueous solutions using an on-demand droplet dispenser (MicroFab MJ-APB-050), injected into the DBQ-EDB through an induction electrode (<±500 V_dc_ typical), and electrostatically confined axially within a vertically oriented quadrupole (±600 V_ac_, 300 Hz typical). Counterbalance electrodes (<±500 V_dc_ typical) countered the force of gravity and the nitrogen gas flow to achieve levitation. The RH inside the levitation chamber was controlled by varying the ratio of dry and humidified nitrogen gas (total flow 500 sccm typical). A 671-nm laser was used for far-field imaging to avoid absorption by the growth media (as seen in *SI Appendix*, Fig. S15), and a blue light-emitting diode (LED) was used for bright-field imaging.

### Dual-Balance Technique.

The experimental process for inferring aerosol viscosity and identifying aerosol gel transitions is described in extensive detail in previous publications (35). In the present study, particles were equilibrated at a constant RH for 10 min and then merged. The coalescence process was tracked with bright-field or far-field laser scatter imaging. For TSB, the coalescence timescale τ (extracted time constant from an exponential fit of aspect ratio as a function of time) was related to viscosity through the relationship τ to ∼(*η·r)/*σ, where η is the viscosity of the aerosols, σ is the surface tension [which was estimated to be ∼55 mN ⋅ m^−1^ (35, 74)], and *r* is the radius of the merged dimer.

### Single-Particle Characterization to Identify Efflorescence.

The voltage necessary to levitate a droplet is directly proportional to particle mass. To identify efflorescence phase transitions (which result in abrupt change in mass because of rapid water loss), we characterized changes in single-particle mass relative to 80% RH, and the relative mass of a particle is calculated as V_dc_(RH)/V_dc_(80% RH), where V_dc_(RH) and V_dc_(80% RH) are the counterbalance voltages necessary to levitate the droplet under zero airflow at, respectively, the set RH and 80% RH (see *SI Appendix*, *SI Methods* for expanded details). Any efflorescence transition could be clearly identified by the abrupt change in mass (as seen in Figs. 2 and 3 and *SI Appendix*, Fig. S1) as well as distinct changes in the far-field laser scatter, as demonstrated in *SI Appendix*, Fig. S2.

### Statistical Analysis.

Each experiment was performed for a minimum of four trials, and respective values reported ±1 SD. Average RH values include propagated uncertainty from individual RH measurements. RH_eff_ values from single-particle measurements are reported as the RH (±1 SD), in which efflorescence was observed. At this reported RH_eff_, the efflorescence probability, *P*_eff_ = *N*_eff_/*N*_tot_, is 1.0, where *N*_eff_ and *N*_tot_ are the number of particles observed to effloresce and the total number of particles, respectively. Uncertainty in *P*_eff_ is estimated as $\pm 1/\sqrt{N_{\mathrm{tot}}}$ (21). See *SI Appendix*, *Factors Influencing Phase Changes* for additional discussion.

### Microscopy.

Evaporating droplets were imaged using a fluorescence microscope (Olympus BX40) equipped with a mercury burner as the excitation light source (U-LH100HG) on both hydrophobic (glass slides coated with a hydrophobic film [Rain-X]) and hydrophilic (uncoated glass slides) surfaces. Droplets were deposited on slides by pipetting 3 µL solution directly on the surface. UV excitation was used to identify the location of proteins and peptides containing aromatic amino acids (see *SI Appendix*, *SI Methods* for expanded details). Droplets were exposed to ambient laboratory air (∼40% RH). For DMEM droplets, it was necessary to blow dry nitrogen across the drop to initiate crystallization.

### Chemicals and Solution Preparation.

All materials were purchased as dry powders through Sigma-Aldrich. DMEM, LB, and TSB solutions were made at 4 wt.% from dry powders in 18 MΩ water that was filtered prior to use (0.45 µm pore size); see *SI Appendix*, Table S1 for the specific formulations of the culture media. Culture media solutions were made fresh daily. Solutions of NaCl (ACS reagent, >99%), CaCl_2_ (ACS reagent, >96%), albumin (from porcine serum, >98%), and mucin (from porcine stomach, type III) were made at 3 to 4 wt.% total solute concentration.

## Supplementary Material

Supplementary File

Supplementary File

## Data Availability

All study data are included in the article and/or supporting information.

## References

[1] J. W. Tang, The effect of environmental parameters on the survival of airborne infectious agents. J. R. Soc. Interface 6 (suppl. 6), S737–S746 (2009). 19773291 10.1098/rsif.2009.0227.focus PMC2843949

[2] K. A. Kormuth , Influenza virus infectivity is retained in aerosols and droplets independent of relative humidity. J. Infect. Dis. 218, 739–747 (2018). 29878137 10.1093/infdis/jiy221 PMC6057527

[3] K. Lin, C. R. Schulte, L. C. Marr, Survival of MS2 and Φ6 viruses in droplets as a function of relative humidity, pH, and salt, protein, and surfactant concentrations. PLoS One 15, e0243505 (2020). 33290421 10.1371/journal.pone.0243505 PMC7723248

[4] S. Asadi , Aerosol emission and superemission during human speech increase with voice loudness. Sci. Rep. 9, 2348 (2019). 30787335 10.1038/s41598-019-38808-z PMC6382806

[5] W. G. Lindsley , Viable influenza A virus in airborne particles expelled during coughs versus exhalations. Influenza Other Respir. Viruses 10, 404–413 (2016). 26991074 10.1111/irv.12390 PMC4947941

[6] P. Fabian , Influenza virus in human exhaled breath: An observational study. PLoS One 3, e2691 (2008). 18628983 10.1371/journal.pone.0002691 PMC2442192

[7] W. Yang, L. C. Marr, Mechanisms by which ambient humidity may affect viruses in aerosols. Appl. Environ. Microbiol. 78, 6781–6788 (2012). 22820337 10.1128/AEM.01658-12 PMC3457514

[8] W. Yang, S. Elankumaran, L. C. Marr, Relationship between humidity and influenza A viability in droplets and implications for influenza’s seasonality. PLoS One 7, e46789 (2012). 23056454 10.1371/journal.pone.0046789 PMC3463543

[9] M. Schuit , The influence of simulated sunlight on the inactivation of influenza virus in aerosols. J. Infect. Dis. 221, 372–378 (2020). 31778532 10.1093/infdis/jiz582

[10] K. Lin, L. C. Marr, Humidity-dependent decay of viruses, but not bacteria, in aerosols and droplets follows disinfection kinetics. Environ. Sci. Technol. 54, 1024–1032 (2020). 31886650 10.1021/acs.est.9b04959

[11] J. Shaman, M. Kohn, Absolute humidity modulates influenza survival, transmission, and seasonality. Proc. Natl. Acad. Sci. U.S.A. 106, 3243–3248 (2009). 19204283 10.1073/pnas.0806852106 PMC2651255

[12] A. J. Prussin II , Survival of the enveloped virus Phi6 in droplets as a function of relative humidity, absolute humidity, and temperature. Appl. Environ. Microbiol. 84, e00551-18 (2018). 29625986 10.1128/AEM.00551-18 PMC5981065

[13] A. Fedorenko, M. Grinberg, T. Orevi, N. Kashtan, Survival of the enveloped bacteriophage Phi6 (a surrogate for SARS-CoV-2) in evaporated saliva microdroplets deposited on glass surfaces. Sci. Rep. 10, 22419 (2020). 33376251 10.1038/s41598-020-79625-z PMC7772334

[14] S. Niazi , Susceptibility of an airborne common cold virus to relative humidity. Environ. Sci. Technol. 55, 499–508 (2021). 33332096 10.1021/acs.est.0c06197

[15] D. H. Morris , Mechanistic theory predicts the effects of temperature and humidity on inactivation of SARS-CoV-2 and other enveloped viruses. eLife 10, e65902 (2021). 33904403 10.7554/eLife.65902 PMC8277363

[16] S. Niazi , Humidity-dependent survival of an airborne influenza A virus: Practical implications for controlling airborne viruses. Environ. Sci. Technol. Lett. 8, 412–418 (2021).

[17] A. I. Donaldson, N. P. Ferris, The survival of some air-borne animal viruses in relation to relative humidity. Vet. Microbiol. 1, 413–420 (1976).

[18] E. P. Vejerano, L. C. Marr, Physico-chemical characteristics of evaporating respiratory fluid droplets. J. R. Soc. Interface 15, 20170939 (2018). 29491178 10.1098/rsif.2017.0939 PMC5832737

[19] I. N. Tang, H. R. Munkelwitz, N. Wang, Water activity measurements with single suspended droplets: The NaCl-H_2_O and KCl-H_2_O systems. J. Colloid Interface Sci. 114, 409–415 (1986).

[20] R. D. Davis, S. Lance, J. A. Gordon, M. A. Tolbert, Long working-distance optical trap for in situ analysis of contact-induced phase transformations. Anal. Chem. 87, 6186–6194 (2015). 25961113 10.1021/acs.analchem.5b00809

[21] R. D. Davis, M. A. Tolbert, Crystal nucleation initiated by transient ion-surface interactions at aerosol interfaces. Sci. Adv. 3, e1700425 (2017). 28776032 10.1126/sciadv.1700425 PMC5517112

[22] D. S. Richards , Ion-molecule interactions enable unexpected phase transitions in organic-inorganic aerosol. Sci. Adv. 6, eabb5643 (2020). 33208357 10.1126/sciadv.abb5643 PMC7673807

[23] E. Mikhailov, S. Vlasenko, S. T. Martin, T. Koop, U. Pöschl, Amorphous and crystalline aerosol particles interacting with water vapor: Conceptual framework and experimental evidence for restructuring, phase transitions and kinetic limitations. Atmos. Chem. Phys. 9, 9491–9522 (2009).

[24] H. P. Dette, T. Koop, Glass formation processes in mixed inorganic/organic aerosol particles. J. Phys. Chem. A 119, 4552–4561 (2015). 25490407 10.1021/jp5106967

[25] A. Marsh , Amorphous phase state diagrams and viscosity of ternary aqueous organic/organic and inorganic/organic mixtures. Phys. Chem. Chem. Phys. 20, 15086–15097 (2018). 29796502 10.1039/c8cp00760h

[26] B. Zobrist , Ultra-slow water diffusion in aqueous sucrose glasses. Phys. Chem. Chem. Phys. 13, 3514–3526 (2011). 21229162 10.1039/c0cp01273d

[27] M. Shiraiwa, M. Ammann, T. Koop, U. Pöschl, Gas uptake and chemical aging of semisolid organic aerosol particles. Proc. Natl. Acad. Sci. U.S.A. 108, 11003–11008 (2011). 21690350 10.1073/pnas.1103045108 PMC3131339

[28] E. Mikhailov, S. Vlasenko, R. Niessner, U. Pöschl, Interaction of aerosol particles composed of protein and saltswith water vapor: Hygroscopic growth and microstructural rearrangement. Atmos. Chem. Phys. 4, 323–350 (2004).

[29] D. J. Stewart , Liquid-liquid phase separation in mixed organic/inorganic single aqueous aerosol droplets. J. Phys. Chem. A 119, 4177–4190 (2015). 25879138 10.1021/acs.jpca.5b01658

[30] V. G. Ciobanu, C. Marcolli, U. K. Krieger, U. Weers, T. Peter, Liquid-liquid phase separation in mixed organic/inorganic aerosol particles. J. Phys. Chem. A 113, 10966–10978 (2009). 19775109 10.1021/jp905054d

[31] A. Tabazadeh, Organic aggregate formation in aerosols and its impact on the physicochemical properties of atmospheric particles. Atmos. Environ. 39, 5472–5480 (2005).

[32] R. E. H. Miles, J. F. Davies, J. P. Reid, The influence of the surface composition of mixed monolayer films on the evaporation coefficient of water. Phys. Chem. Chem. Phys. 18, 19847–19858 (2016). 27388102 10.1039/c6cp03826c

[33] J. F. Davies, A. E. Haddrell, R. E. H. Miles, C. R. Bull, J. P. Reid, Bulk, surface, and gas-phase limited water transport in aerosol. J. Phys. Chem. A 116, 10987–10998 (2012). 23095147 10.1021/jp3086667

[34] N. G. Di Novo, A. R. Carotenuto, G. Mensitieri, M. Fraldi, N. M. Pugno, Modeling of virus survival time in respiratory droplets on surfaces: A new rational approach for antivirus strategies. Front. Mater. 8, 631723 (2021).

[35] D. S. Richards, K. L. Trobaugh, J. Hajek-Herrera, R. D. Davis, Dual-balance electrodynamic trap as a microanalytical tool for identifying gel transitions and viscous properties of levitated aerosol particles. Anal. Chem. 92, 3086–3094 (2020). 31941272 10.1021/acs.analchem.9b04487

[36] R. D. Davis, S. Lance, J. A. Gordon, S. B. Ushijima, M. A. Tolbert, Contact efflorescence as a pathway for crystallization of atmospherically relevant particles. Proc. Natl. Acad. Sci. U.S.A. 112, 15815–15820 (2015). 26668396 10.1073/pnas.1522860113 PMC4702978

[37] F. W. Teale, G. Weber, Ultraviolet fluorescence of the aromatic amino acids. Biochem. J. 65, 476–482 (1957). 13412650 10.1042/bj0650476 PMC1199900

[38] A. Bortolotti , On the purported “backbone fluorescence” in protein three-dimensional fluorescence spectra. RSC Adv. 6, 112870–112876 (2016).

[39] T. A. Wani, A. H. Bakheit, S. Zargar, M. A. Hamidaddin, I. A. Darwish, Spectrophotometric and molecular modelling studies on in vitro interaction of tyrosine kinase inhibitor linifanib with bovine serum albumin. PLoS One 12, e0176015 (2017). 28419132 10.1371/journal.pone.0176015 PMC5395234

[40] K. L. Zapadka, F. J. Becher, A. L. Gomes Dos Santos, S. E. Jackson, Factors affecting the physical stability (aggregation) of peptide therapeutics. Interface Focus 7, 20170030 (2017). 29147559 10.1098/rsfs.2017.0030 PMC5665799

[41] R.-N. Liu, Y.-M. Kang, Stochastic master equation for early protein aggregation in the transthyretin amyloid disease. Sci. Rep. 10, 12437 (2020). 32709875 10.1038/s41598-020-69319-x PMC7381670

[42] O. W. Meldrum , Mucin gel assembly is controlled by a collective action of non-mucin proteins, disulfide bridges, Ca^2+^-mediated links, and hydrogen bonding. Sci. Rep. 8, 5802 (2018). 29643478 10.1038/s41598-018-24223-3 PMC5895598

[43] M. Kastelic, Y. V. Kalyuzhnyi, B. Hribar-Lee, K. A. Dill, V. Vlachy, Protein aggregation in salt solutions. Proc. Natl. Acad. Sci. U.S.A. 112, 6766–6770 (2015). 25964322 10.1073/pnas.1507303112 PMC4450416

[44] A. C. Dumetz, A. M. Chockla, E. W. Kaler, A. M. Lenhoff, Protein phase behavior in aqueous solutions: Crystallization, liquid-liquid phase separation, gels, and aggregates. Biophys. J. 94, 570–583 (2008). 18160663 10.1529/biophysj.107.116152 PMC2157236

[45] T. Koop, J. Bookhold, M. Shiraiwa, U. Pöschl, Glass transition and phase state of organic compounds: Dependency on molecular properties and implications for secondary organic aerosols in the atmosphere. Phys. Chem. Chem. Phys. 13, 19238–19255 (2011). 21993380 10.1039/c1cp22617g

[46] H. Y. Reynolds, J. Chrétien, Respiratory tract fluids: Analysis of content and contemporary use in understanding lung diseases. Dis. Mon. 30, 1–103 (1984). 10.1016/0011-5029(84)90008-7 6363022

[47] S. S. Spicer, J. R. Martinez, Mucin biosynthesis and secretion in the respiratory tract. Environ. Health Perspect. 55, 193–204 (1984). 6376099 10.1289/ehp.8455193 PMC1568380

[48] S. Niazi, R. Groth, K. Spann, G. R. Johnson, The role of respiratory droplet physicochemistry in limiting and promoting the airborne transmission of human coronaviruses: A critical review. Environ. Pollut. 276, 115767 (2021). 33243541 10.1016/j.envpol.2020.115767 PMC7645283

[49] M. Hassoun, P. G. Royall, M. Parry, R. D. Harvey, B. Forbes, Design and development of a biorelevant simulated human lung fluid. J. Drug Deliv. Sci. Technol. 47, 485–491 (2018). 30283501 10.1016/j.jddst.2018.08.006 PMC6156579

[50] M. R. Fries , Enhanced protein adsorption upon bulk phase separation. Sci. Rep. 10, 10349 (2020). 32587383 10.1038/s41598-020-66562-0 PMC7316800

[51] W. M. Babinchak, W. K. Surewicz, Liquid-liquid phase separation and its mechanistic role in pathological protein aggregation. J. Mol. Biol. 432, 1910–1925 (2020). 32169484 10.1016/j.jmb.2020.03.004 PMC7395662

[52] M. M. Castellanos, J. A. Pathak, R. H. Colby, Both protein adsorption and aggregation contribute to shear yielding and viscosity increase in protein solutions. Soft Matter 10, 122–131 (2014). 24651563 10.1039/c3sm51994e

[53] J. F. Davies, C. L. Price, J. Choczynski, R. K. Kohli, Hygroscopic growth of simulated lung fluid aerosol particles under ambient environmental conditions. Chem. Commun. (Camb.) 57, 3243–3246 (2021). 33646231 10.1039/d1cc00066g

[54] S. B. Ushijima, R. D. Davis, M. A. Tolbert, Immersion and contact efflorescence induced by mineral dust particles. J. Phys. Chem. A 122, 1303–1311 (2018). 29332388 10.1021/acs.jpca.7b12075

[55] C. L. Price, A. Bain, B. J. Wallace, T. C. Preston, J. F. Davies, Simultaneous retrieval of the size and refractive index of suspended droplets in a linear quadrupole electrodynamic balance. J. Phys. Chem. A 124, 1811–1820 (2020). 32013433 10.1021/acs.jpca.9b10748

[56] J. Vörös, The density and refractive index of adsorbing protein layers. Biophys. J. 87, 553–561 (2004). 15240488 10.1529/biophysj.103.030072 PMC1304376

[57] M. O. Fernandez, R. J. Thomas, H. Oswin, A. E. Haddrell, J. P. Reid, Transformative approach to investigate the microphysical factors influencing airborne transmission of pathogens. Appl. Environ. Microbiol. 86, e01543-20 (2020). 32978136 10.1128/AEM.01543-20 PMC7657628

[58] N. Zhu , China Novel Coronavirus Investigating and Research Team, A novel coronavirus from patients with pneumonia in China, 2019. N. Engl. J. Med. 382, 727–733 (2020). 31978945 10.1056/NEJMoa2001017 PMC7092803

[59] S. Torkzaban, S. M. Hassanizadeh, J. F. Schijven, H. H. J. L. van den Berg, Role of air-water interfaces on retention of viruses under unsaturated conditions. Water Resour. Res. 42, 1–11 (2006).

[60] T. Trouwborst, S. Kuyper, J. C. de Jong, A. D. Plantinga, Inactivation of some bacterial and animal viruses by exposure to liquid-air interfaces. J. Gen. Virol. 24, 155–165 (1974). 4367307 10.1099/0022-1317-24-1-155

[61] J. F. Davies, K. R. Wilson, Raman spectroscopy of isotopic water diffusion in ultraviscous, glassy, and gel states in aerosol by use of optical tweezers. Anal. Chem. 88, 2361–2366 (2016). 26751163 10.1021/acs.analchem.5b04315

[62] H. A. Kramers, Brownian motion in a field of force and the diffusion model of chemical reactions. Physica 7, 284–304 (1940).

[63] G. Gadda, P. Sobrado, Kinetic solvent viscosity effects as probes for studying the mechanisms of enzyme action. Biochemistry 57, 3445–3453 (2018). 29874467 10.1021/acs.biochem.8b00232

[64] L. S. Ulanova , Development of methods for encapsulation of viruses into polymeric nano- and microparticles for aquaculture vaccines. J. Appl. Polym. Sci. 131, 8785–8796 (2014).

[65] V. Kaushik, Y. H. Roos, Lipid encapsulation in glassy matrices of sugar-gelatin systems in freeze-drying. Int. J. Food Prop. 11, 363–378 (2008).

[66] Z. Zuo , Survival of airborne MS2 bacteriophage generated from human saliva, artificial saliva, and cell culture medium. Appl. Environ. Microbiol. 80, 2796–2803 (2014). 24561592 10.1128/AEM.00056-14 PMC3993287

[67] G. J. Harper, Airborne micro-organisms: Survival tests with four viruses. J. Hyg. (Lond.) 59, 479–486 (1961). 10.1017/s0022172400039176 PMC2134455 13904777

[68] D. B. Hill , A biophysical basis for mucus solids concentration as a candidate biomarker for airways disease. PLoS One 9, e87681 (2014). Correction in: *PLoS One* **9**, e97980 (2014). 24558372 10.1371/journal.pone.0087681 PMC3928107

[69] H. P. Oswin , Measuring stability of virus in aerosols under varying environmental conditions. Aerosol Sci. Technol. 55, 1315–1320 (2021).

[70] G. E. Hatch, “Comparative biochemistry of airway lining fluid” in Comparative Biology of the Normal Lung, Vol. 1: Treatise on Pulmonary Toxicology, R. Parent, Ed. (CRC Press, Inc., 1992), pp. 617–632.

[71] J. Yan , EMIT Consortium, Infectious virus in exhaled breath of symptomatic seasonal influenza cases from a college community. Proc. Natl. Acad. Sci. U.S.A. 115, 1081–1086 (2018). 29348203 10.1073/pnas.1716561115 PMC5798362

[72] R. Groth, L. T. Cravigan, S. Niazi, Z. Ristovski, G. R. Johnson, In situ measurements of human cough aerosol hygroscopicity. J. R. Soc. Interface 18, 20210209 (2021). 33947221 10.1098/rsif.2021.0209 PMC8097516

[73] G. R. Johnson , Modality of human expired aerosol size distributions. J. Aerosol Sci. 42, 839–851 (2011).

[74] D. R. Absolom, C. J. Van Oss, W. Zingg, A. W. Neumann, Determination of surface tensions of proteins. II. Surface tension of serum albumin, altered at the protein-air interface. Biochim. Biophys. Acta 670, 74–78 (1981). 7272330 10.1016/0005-2795(81)90050-7

