# Supplementary material for: Evidence for a semisolid phase state of aerosols and droplets relevant to the airborne and surface survival of pathogens
Source: Proc Natl Acad Sci U S A. 2022 Jan 21;119(4):e2109750119. doi: 10.1073/pnas.2109750119 (PMC8794803; doi:10.1073/pnas.2109750119)
Supplement: Supplementary File [file pnas.2109750119.sapp.pdf]

Supplementary information for

Evidence for a semi-solid phase state of aerosols and droplets relevant to the airborne and surface survival of pathogens

Erik Huynh,[1,†] Anna Olinger,[1,†] David Woolley,[1] Ravleen Kaur Kohli,[2] Jack M. Choczynski,[2] James F. Davies,[2] Kaisen Lin,[3,‡] Linsey C. Marr,[3] Ryan D. Davis[1]*

[1]Department of Chemistry, Trinity University, San Antonio, TX 78212, USA.
[2]Department of Chemistry, University of California-Riverside, Riverside, CA 92521, USA.
[3]Department of Civil and Environmental Engineering, Virginia Tech, Blacksburg, VA, USA.

*Corresponding author: rdavis5@trinity.edu
[†]Equal contribution
[‡]Present address: Air Quality Research Center, University of California- Davis, Davis, CA, 95616, USA

Table of contents:

Page S-2: Supplemental Methods
Page S-4: Factors Influencing Phase Changes
Page S-5: Effects of Diffusive Limitations
Page S-6: Discussion of Temperature Effects
Page S-7: Fig. S1. Mass growth factors for single-particle characterization
Fig. S2. Characteristic bright-field and far-field images of levitated aerosol particles
Page S-8: Fig. S3. Dual-particle coalescence plots (aspect ratio as a function of time) for various trials of merging levitated aerosol particles
Page S-9: Fig. S4. Images of merged aerosols ejected from the DBQ-EDB
Page S-10: Fig. S5. The phase state of aerosols as a function of conditioning time
Fig. S6. An example of contact efflorescence of NaCl-protein mixtures initiated upon merging
Page S-11: Fig. S7. Mie resonance measurements of 1:1 NaCl:protein particles
Page S-12: Fig. S8. Efflorescence of merged, rigid NaCl-$CaCl_2$-protein aerosol with decreasing RH
Page S-13: Fig. S9. Microscopy images of evaporating droplets on hydrophobic surfaces
Page S-14: Fig. S10. Microscopy images of evaporating droplets on hydrophilic surfaces
Page S-15: Fig. S11. Line profiles of evaporating drops showing phase separation
Page S-16: Fig. S12. Morphology of acoustically-levitated NaCl-protein droplets
Page S-17: Fig. S13. The morphology of lysogeny broth (LB) droplets with and without E. coli.
Fig. S14. Partitioning of fluorescent PSLs
Page S-18: Fig. S15. Visible absorbance spectra of growth media
Page S-19: Table S1. Composition of growth media
Page S-20: Table S2. Initial contact angles of droplets on glass slides
Caption to Movie S1
Supplemental References

Supplemental Methods

Aerosol levitation. Aerosol particles were levitated in a dual-balance quadrupole electrodynamic balance (DBQ-EDB), as described extensively elsewhere (1). The DBQ-EDB was used for both single-particle and dual-particle characterization. Aerosol particles are generated from aqueous solutions using an on-demand droplet dispenser (MicroFab MJ-APB-050) and injected into the DBQ-EDB through an induction electrode (< ±500 V_{dc} typical), which induces a charge on the surface of the particle. The magnitude and polarity of the charge on the droplet depended on which experimental arrangement was utilized (single- or dual-particle characterization, as described in their respective sections). Charged droplets were electrostatically-confined axially within a vertically-oriented quadrupole (±600 V_{ac}, 300 Hz typical). Counterbalance electrodes (<±500 V_{dc} typical) countered the force of gravity and the nitrogen gas flow to achieve levitation.

The RH inside the levitation chamber was controlled by varying the ratio of dry and humidified nitrogen gas (total flow 500 sccm typical). The RH was measured with probes placed at the inlet and outlet of the chamber, and the RH inside the chamber was determined as the average of the two probe readings (±1 SD). All experiments were conducted at room temperature (295±2 K).

Aerosol imaging. Levitated aerosols were primarily imaged with a bright-field arrangement. Far-field laser scatter imaging was also used intermittently to confirm the morphology of the levitated aerosols (see Fig. S2 for examples). For bright-field imaging, levitated aerosols were backlit with a blue or red LED and imaged with a 20X long working-distance microscope objective and CMOS camera. Images were recorded and analyzed in a custom LabVIEW program. Levitated particles were sized with the bright-field arrangement by calibrating the collected images. Far-field images were collected using a 671 nm diode-pumped laser and imaging the far-field elastically-scattered light with a CMOS camera.

Dual-balance technique to identify amorphous phase states and infer viscosity. The experimental process for inferring aerosol viscosity and identifying aerosol gel transitions is described in extensive detail in previous publications (1).The DBQ-EDB levitates two oppositely-charged particles ~5 cm apart at a set RH value. In the present study, particles were equilibrated at a constant RH for 10 minutes and then merged. The coalescence process was tracked with bright-field or far-field laser scatter imaging. The RH-dependent viscosity or amorphous state of model respiratory compounds and growth media aerosol was studied with this electrostatic levitation technique.

To infer viscosity and amorphous phase state, the bright-field images were binarized and the aspect ratio of the merged dimer was tracked as a function of time, as illustrated in Fig. 1, with plots shown in Fig. S3. For non-viscous fluids, complete coalescence to a final aspect ratio of unity occurred more rapidly than our imaging system can resolve (<0.1 s, corresponding to a viscosity less than ~300 Pa·s). With decreasing RH, it was observed that many aerosol compositions would not coalesce completely (within an observation time of at least 10 min), and the aspect ratio of merged dimers remained greater than 1, as shown in Fig. S3. This was indicative of a semi-solid state, where the particles were partially fluid (which allowed for partial coalescence) and partially solid or semi-solid (which inhibited complete coalescence). In this scenario, it was not possible to accurately measure viscosity since the particle was non-uniform. Thus, when there was incomplete coalescence, rather than characterizing semi-solid states on the basis of viscosity, the RH at which the aspect ratio of merged dimers was consistently greater than ~1.1 was taken as the RH at which a semi-solid transition occurred.

For TSB aerosols, it was possible to measure the viscosity across a wide RH range (~40-75% RH) because complete coalescence was observed, with coalescence timescales (τ) ranging from 0.1-400 s. Coalescence timescale is the extracted time constant from an exponential fit of aspect ratio as a function of time. The time constant is related to viscosity through the relationship $\tau \approx (\eta \cdot r)/\sigma$, where η is the viscosity of the aerosols, σ is the surface tension (which was estimated to be 55 mN·m^{-1}, on the basis of past studies and the surface tension of protein solutions (2, 3)), and r is the radius of the relaxed sphere

(1). At RH < 40%, TSB was completely rigid and did not coalesce, and the lower-limit for viscosity was estimated from the observation time over which merged dimers were observed (~7200 s).

Single-particle characterization to identify efflorescence. The voltage necessary to levitate an aerosol particle is directly proportional to particle mass. Here, to identify efflorescence phase transitions (which result in an abrupt change in mass due to rapid water loss) we characterized changes in single-particle mass relative to 80% RH, and the relative mass of a particle is calculated as V_{dc}(RH)/V_{dc}(80% RH), where V_{dc}(RH) and V_{dc}(80% RH) are the counterbalance voltages necessary to levitate the droplet under zero air flow at, respectively, the set RH and 80% RH. In Figs. 2 and 3, data is presented in terms of relative mass loss to be explicit about how experiments were performed; for data in terms of mass growth factors, see Fig. S1.

To perform a trial, a single particle is trapped at ~80% RH and equilibrated for 10 min. The gas flow is momentarily switched off (<3 s) to measure V_{dc}(80% RH). The RH was reduced by 5-7%, and V_{dc}(RH) was measured after re-equilibrating for 10 min at the lower RH. During this process, bright-field images we continuously collected. The laser was turned on to briefly collect far-field images at each RH after measuring V_{dc}(RH). Thus, any efflorescence transition could be clearly identified by the abrupt change in mass (as seen in Figs. 2 and 3 and S1) as well as distinct changes in the far-field laser scatter, as demonstrated in Fig. S2.

Microscopy. Evaporating droplets were imaged using a fluorescence microscope (Olympus BX40) equipped with a mercury burner as the excitation light source (U-LH100HG). Droplets were deposited on slides by pipetting 3 μL of solution directly on the surface. UV-excitation was used to identify the location of proteins and peptides containing aromatic amino acids. A shortpass filter (cut-off wavelength of 400 nm) passed the excitation light, while a long-pass filter with a cut-on wavelength of 450 nm passed emitted light to the imaging system. The emission maximum for albumin is ~350 nm, while light >450 nm is at the tail end of the emission spectrum for albumin (4). Thus, images appear blue, despite the maximum emission occurring at lower wavelengths.

Droplets were exposed to ambient laboratory air (~40% RH). For DMEM droplets, it was necessary to blow dry nitrogen across the drop to initiate crystallization. Evaporating droplets were studied on both hydrophobic (glass slides coated with a hydrophobic film) and hydrophilic (uncoated glass slides) surfaces. The hydrophobic film was applied using a commercially-available treatment (Rain-X).

For droplets, contact angle measurements were conducted with a horizontal microscope arrangement. An example of a horizontally-collected image is shown in Fig. S10K. Images were processed in a custom LabVIEW program using the Vision Assistant software package.

In addition to imaging evaporating droplets, microscopy was used to study the morphology of levitated aerosols after merging. These studies helped confirm the inferred morphologies from the dual-balance characterization in the DBQ-EDB. Merged aerosols were ejected from the DBQ-EDB and collected onto a microscope slide for analysis. During these studies, the ambient laboratory air varied from 50-65% RH, with daily variations due to changes in local weather conditions. See figure captions for the specific RH at which an image was collected after ejection from the trap.

Mie-resonance spectroscopy of singly-levitated particles. Levitated aerosols (~5 μm diameter) in a linear quadrupole electrodynamic balance were illuminated with broadband light from an LED and the backscattered spectrum was measured (5). For homogeneous spherical particles, a well-defined resonance structure is observed that is used to determine the size and optical properties of the particle. In the case of phase-separated particles, if they remain spherical, a resonance structure will be observed, but may be difficult to interpret if a homogeneous composition is assumed (6). If the particle deviates from a spherical morphology, the resonance peaks broaden and are eventually quenched. For highly non-spherical shapes, such as those exhibited by effloresced particles, the resonance structure is fully lost.

Factors Influencing Phase Changes.

A primary consideration for a phase transition is the solute concentration and water content of the particle phase. For example, the crystalline phase of NaCl is not thermodynamically stable above a water activity of ~0.75 at 298 K. Thus, for efflorescence to occur, aqueous NaCl must be at equilibrium with an RH less than 75%. Similarly, many other transitions have a finite range of solute concentrations over which they can occur. Gelation and phase separation, including both liquid-liquid phase separation and aggregation, often have a critical solute concentration, and thus water content, at which they occur (6–8). By contrast, the process of thickening and vitrification can be described as a gradual process occurring across a wider range of conditions (9). However, for all states of matter, there are factors beyond solute concentration that influence the probability and timescale of a phase transition.

Efflorescence is a nucleation-initiated process and is typically described as stochastic (10). That is, efflorescence has a finite probability of occurring across a range of RH values and experimental conditions. For example, with the case of NaCl efflorescence, there is a finite probability of efflorescence occurring at any RH below 75%, i.e., any RH where NaCl is supersaturated. Under most conditions, the probability of efflorescence at such a high RH is negligible due to the kinetic limitations associated with forming a critical crystal nucleus (10). Thus, even though the crystalline phase of NaCl may be thermodynamically stable at an equilibrium RH of 75%, homogeneous efflorescence does not have a significant probability until much lower RH, e.g., ~45% RH for NaCl. The probability of efflorescence at higher RH increases when considering heterogeneous nucleation due to the presence of surfaces that can stabilize a critical nucleus (10, 11). For a set of given experimental conditions, the efflorescence probability, P_{eff}, can be quantified as $P_{\mathrm{eff}} = N_{\mathrm{eff}}/N_{\mathrm{tot}}$, where N_{eff} and N_{tot} are the number of particles observed to effloresce and the total number of particles observed, respectively. Uncertainty in P_{eff} is estimated as $\pm 1/\sqrt{N_{tot}}$ (11).

For homogeneous efflorescence of levitated aerosols, P_{eff} for a given observation time and set of conditions tends to increase from $P_{\mathrm{eff}} = 0.0$ to 1.0 across a range of ~2-3% RH (11). Most experiments consider observation times of minutes. However, with increasing particle size, i.e., up to large droplets, and at longer timescales, P_{eff} may become non-negligible even for NaCl at 50% RH. Both increasing size and observation time may increase P_{eff} because the probability of observing the stochastic event of nucleation is generally thought to scale with volume and time (10). In the present study, RH_{eff} was reported from single-particle measurements with an observation time of ~10 min at each RH increment, and was the average RH at which all aerosols studied were observed to effloresce. Thus, at this RH, $P_{\mathrm{eff}} = 1.0 \pm 0.4$ for a 10 min observation time. As noted earlier, P_{eff} tends to increase rapidly across a narrow RH range. This can be seen in that the uncertainty in RH_{eff} was typically ±2-3% RH, which is nominally higher than the typical uncertainty in individual RH measurements (±1-2% RH typical). In rotating drum measurements with thousands of aerosol particles, there may be a non-zero P_{eff} above this RH.

Heterogeneous efflorescence, where a surface facilitates nucleation, can occur at higher RH than homogeneous efflorescence (10). Heterogeneous efflorescence may become significant for droplets deposited on a surface, particularly if care is not taken to avoid heterogeneous nucleation. To establish a comprehensive, quantitative understanding of pathogen viability, it will be prudent to understand the phase changes that can occur on a range of different substrates.

Similar considerations apply to the establishment of amorphous phase states. As demonstrated in Fig. S5, there is a time-dependence to the formation of amorphous phase states. This time generally increases with particle size due to increasing timescales associated with evaporation and spatial gradients in viscosity, as discussed further in *SI, Effects of Diffusive Limitations*. Particle composition, the rate of initial evaporation, and drying trajectory can all influence the existence of spatial gradients in amorphous phases (12). Furthermore, the presence of interfaces can influence protein aggregation (13). Thus, surface effects may also be important for the establishment of amorphous phase states. In the present study, we observed some morphological differences in the evaporated morphology of droplets deposited on hydrophobic and hydrophilic glass surfaces.

All of these factors point to the need to consider both particle size and substrate, in addition to composition, when linking particle phase state to pathogen viability. In the present study, we compare our

observations to viability measurements that were performed at timescales that were long relative to evaporation timescales. When interpreting other laboratory observations, such timescales need to be considered as the assumption of quasi-equilibrium may not be valid.

There are additional factors that influence phase transitions beyond those discussed here, including temperature, ion-specific (Hofmeister) effects, and solute-solute interactions (8), which are beyond the scope of this manuscript.

Effects of Diffusive Limitations.

Viscosity can influence the rate of chemical reactions (14) and evaporation of solvent (15) by hindering molecular diffusion. Diffusion coefficients (*D*) for solutes can be estimated from viscosity (η) using the Stokes-Einstein relationship, as given by equation S1

$$D = \frac{kT}{6\pi\eta r_s} \qquad \text{S1}$$

where *k* is the Boltzmann constant, *T* is temperature, and r_s is the radius of the diffusing species (9). With increasing viscosity, the Stokes-Einstein equation predicts a decrease in *D*, i.e., slowed molecular diffusion. Although predictions from Stokes-Einstein become inaccurate at high viscosity, the qualitative trend of decreasing *D* with increasing viscosity is expected (9).

Rates of multi-component chemical reactions, which rely on diffusion of solutes, thus tend to be inversely proportional to viscosity due to decreasing diffusion rates. That is, as viscosity increases, the rate of chemical reactions is expected to decrease (14, 16, 17). It has been suggested that viral disinfection proceeds by exposure to solutes in the surrounding medium (18). In this scenario, increasing viscosity will hinder diffusion of reactants to reactive sites on the surface of the virus, which may slow the disinfection reaction and lead to an increase in viability at high viscosity. We note that intramolecular processes, such as protein folding, can also be sensitive to solvent viscosity, which can influence the stability of certain protein conformations (17).

Diffusive limitations imposed by solution viscosity also leads to long timescales for mixing of solutes. Diffusive mixing timescales (τ_d), i.e., the characteristic time of mass-transport and mixing by molecular diffusion, can be estimated from *D* and equation S2:

$$\tau_d = \frac{a^2}{\pi^2 D} \qquad \text{S2}$$

where *a* is the radius of the aerosol particle or other compartment (19). In the context of disinfection kinetics, the timescale of diffusive mixing can give an estimate for how long it would take a gas-phase disinfectant molecule to become uniformly mixed throughout the particle bulk following gas-to-particle partitioning. For particle-phase reactions, long diffusive timescales are indicative of slow molecular diffusion and hindered reaction kinetics (14, 16). Estimated mixing timescales are reported for a wide range of viscosities and particle diameters in Koop et al. (9) and Shiraiwa et al. (19). Here, we make estimates for micron-sized aerosols, as used in Lin and Marr (18). For a solute with a molecular radius r_s = 1 nm in a micron-sized aerosol (a = 1 μm) and a viscosity of 10^3 Pa·s, τ_d is estimated to be ~8 minutes from equations S1 and S2 for T = 298 K. This timescale is comparable to the time of viability experiments in Lin and Marr (60 minutes) (18). At a viscosity of 10^4 Pa·s, τ_d is estimated to be ~80 minutes, which is longer than the timescale of the viability studies. These timescales are in contrast to mixing that would occur in a low-viscosity solution, such as water, where mixing would occur in less than a millisecond at a viscosity of 10^{-3} Pa·s. Small solutes, such as atomic ions, diffuse more quickly. For an ionic radius of r_s = 0.15 nm, τ_d is estimated to be ~1.1 and 11 minutes at a viscosity of 10^3 and 10^4 Pa·s, respectively. Pathogens are orders-of-magnitude larger in size than solutes implicated in inactivation (e.g., ozone, Na^+), and the resulting diffusion coefficients are thus orders of magnitude lower than smaller molecular and ionic solutes.

Diffusive mixing in droplets occurs at even longer timescales. Thus, for droplets, τ_d, becomes comparable to the timescale of an experiment at lower viscosity (higher RH). This may be one reason that the minimum viability of phi-6 in TSB was at higher RH in droplets (~85%) compared to aerosols (~75%). However, more work remains to be done to identify all the ways that viscosity and diffusive

limitations will influence viral viability. Further, we note that droplets evaporate to a fraction of their original thickness, as seen in Fig. S10K. Thus, due to spreading of the droplet on a surface, diffusive mixing timescales for gas-to-particle partitioning will likely be shorter than if a spherical shape is assumed for droplets.

Discussion of Temperature Effects.

The protective effect of an amorphous semi-solid state may also help explain the temperature dependence of pathogen viability, where viability tends to increase with decreasing temperature (20, 21). Considering NaCl efflorescence RH is quite insensitive to temperature (22), efflorescence alone is inadequate to fully explain enhanced viral survival at low temperatures. Indeed, virions are typically more stable at lower temperatures, irrespective of RH (23). As discussed in Morris et al. (21), enhanced pathogen survival at lower temperatures is expected based on the typical Arrhenius-like behavior of chemical kinetics, resulting in a decreased rate of disinfection. However, a simple Arrhenius model does not capture the effect of temperature-dependent medium viscosity (24, 25). As discussed earlier, viscous states can hinder molecular diffusion and are expected to inhibit chemical reactions, including those involved in disinfection. This is a potentially important consideration because, in contrast to NaCl efflorescence, amorphous phase transitions are highly sensitive to temperature and can occur at higher RH (higher water content) at colder temperatures (26, 27). That is, lower temperatures favor the formation of viscous, gelatinous, and glassy phase states. Thus, temperature is likely influencing disinfection kinetics by modifying the viscosity of the reaction medium, which suggests disinfection kinetics may be over-predicted (and pathogen lifetime under-predicted) for higher RH values at lower temperatures if amorphous phase changes and temperature-dependent viscosity are not considered.

Considering that amorphous phase states can exist at higher RH for lower temperatures, the typical U-shaped survival of viruses would be less pronounced if amorphous phase states were hindering inactivation. Prussin et al. (20) indeed observed a constant RH-dependent survival for phi-6 in tryptic soy broth at 14 °C, in contrast to the U-shaped RH-dependent survival at 25 and 37 °C. Similarly, in Morris et al. (21), the U-shaped survival of SARS-CoV-2 in DMEM droplets was less pronounced at 10 °C than at 22 °C. In that study, the half-life of the virus at 10 °C was comparable at both 65% and 85% RH, in contrast to warmer temperatures (22 and 27 °C) where survival was significantly lower at 65% RH. These observations suggest a link between temperature-dependent particle-phase viscosity and viral survival, where lower temperatures will broaden the RH range over which pathogens can remain viable. Future studies will need to characterize the temperature-dependent phase behavior of growth media and respiratory emissions to confirm a link between temperature, phase state, and pathogen viability.

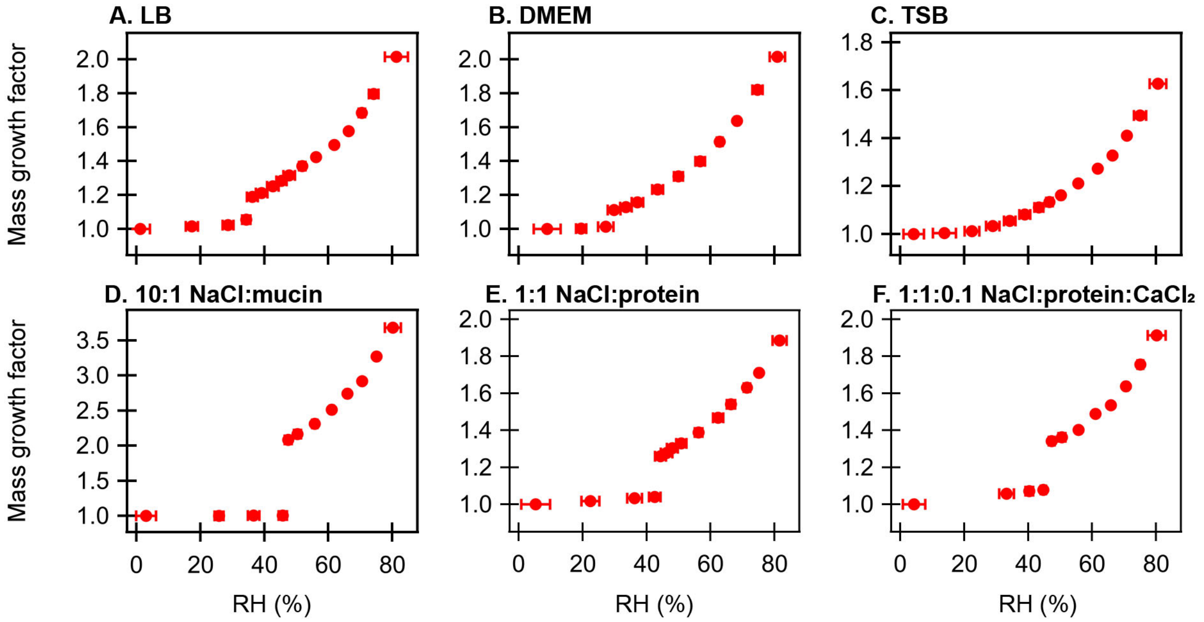

Figure S1. The single-particle data from Figs. 2 and 3 of the main manuscript shown in terms of mass growth factor, where mass growth factor is the mass of the levitated particle as a function of RH relative to the dry mass of the particle. Here, this was calculated as $V(RH)/V(RH \approx 0\%)$, where $V(RH)$ was the counterbalance voltage at a set RH, and $V(RH \approx 0\%)$ was the counterbalance voltage necessary to levitate a particle under arid conditions.

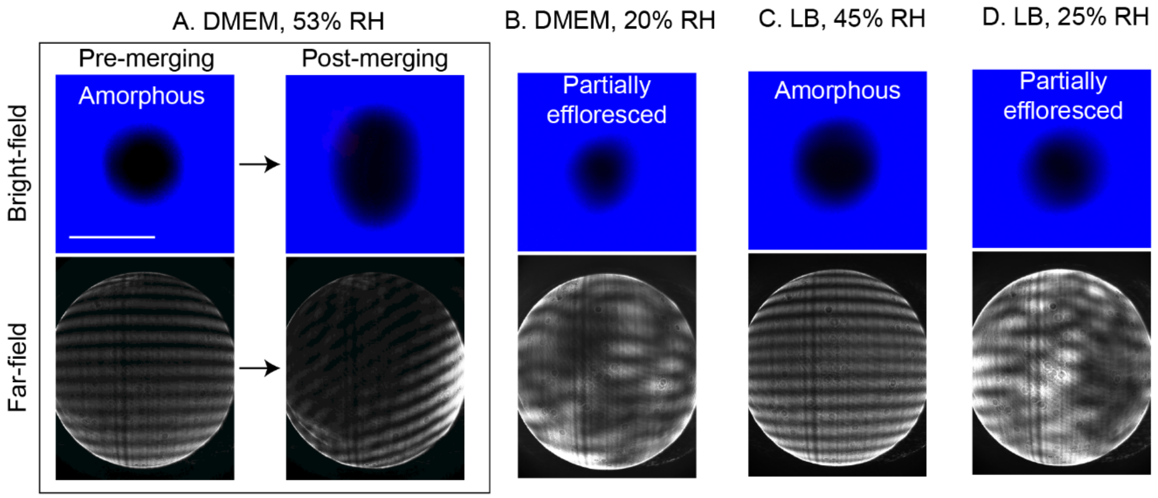

Figure S2. Characteristic bright-field and far-field images associated with different morphologies of levitated aerosol particles. **(A)** Levitated DMEM in an amorphous phase at 53% RH. The first frame (left) shows a single levitated aerosol particle. The bright-field shows a spherical morphology. The far-field image similarly shows linear interference fringes, indicating a spherical shape. The second frame (right) shows the same DMEM particle after merging with a second DMEM particle. The bright-field images show that at 53% RH, the particles have only partially coalesced, as evident from the oblong shape of the merged dimer, indicative of a semi-solid state. The far-field image also confirms a non-spherical shape due to disruptions in the far-field fringes. **(B)** A partially-effloresced DMEM particle at 20% RH. Efflorescence is unambiguous in the far-field images, where the crystal entirely disrupts the interference fringes. **(C)** Levitated LB aerosol at 45% RH, showing an amorphous, non-crystalline state. **(D)** LB after efflorescence at 25% RH.

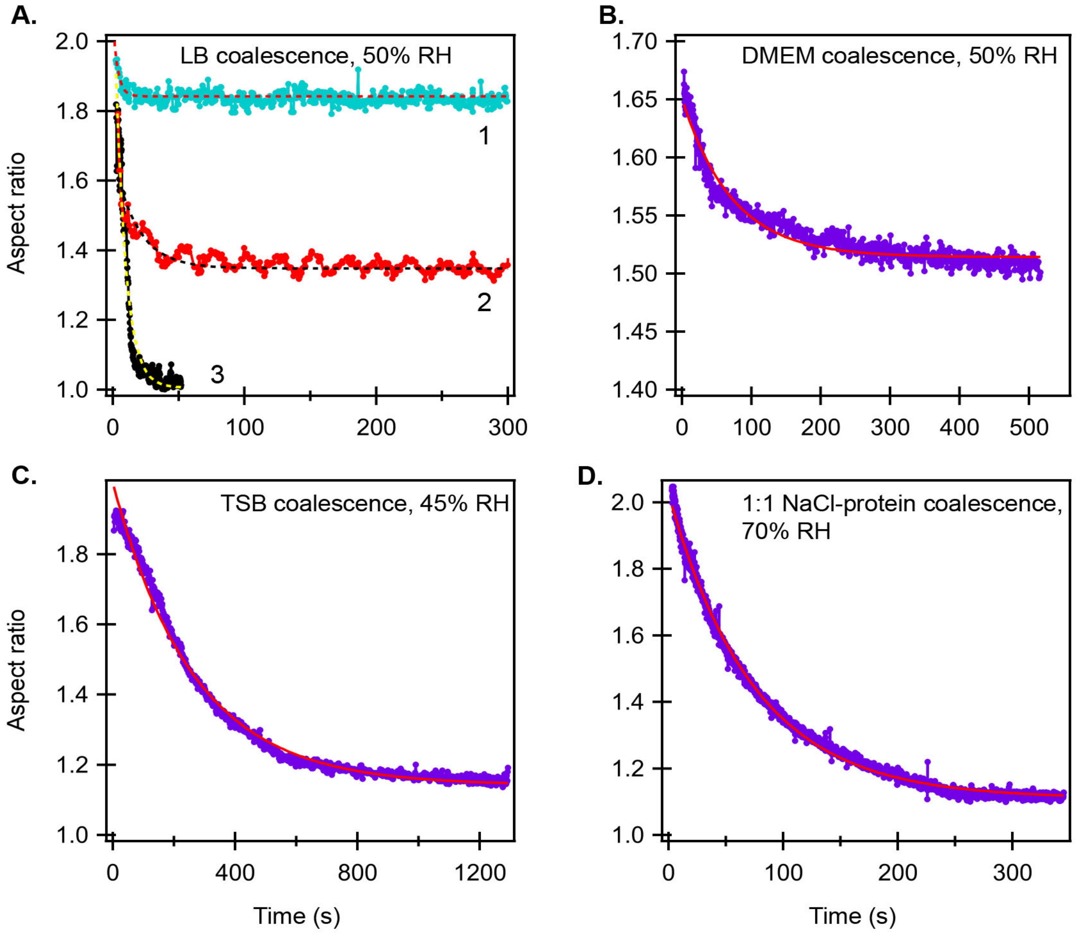

Figure S3. Examples of aspect ratio as a function of time for dual-particle characterization, where levitated aerosols are merged to determine their phase. Lines show exponential fits. **(A)** Semi-solid LB aerosol at 50% RH. Three examples are shown for different trials, demonstrating some particle-to-particle variability. In example 1, the final aspect ratio of the merged dimer is 1.85 (as determined through an exponential fit), indicating a large extent of solid behavior. In example 2, partial coalescence was observed, where the final aspect ratio was 1.35, demonstrating both viscous fluid and amorphous solid properties. In example 3, complete coalescence was observed (final aspect ratio of 1.00), where τ is 7.7 s (equivalent to a viscosity of ~2×10^4 Pa·s), demonstrating a highly viscous fluid state. In examples 1 and 2, the initial coalescence follows a similar timescale as example 3, suggesting the fluid phase has a similar viscosity in all cases. The lack of further coalescence in examples 1 and 2 suggests a rigid phase with a viscosity equivalent to >10^8 Pa s. (We note that inferring viscosity for mixed-phase aerosols using coalescence measurements is more uncertain than for homogeneously-mixed aerosols.) Out of 15 total trials, only one resulted in complete coalescence (example 3); the dominant outcome was partial coalescence, indicative of a rigid, organic-dense phase. The extent of partial coalescence likely reflects some variability in the size, number, and packing of the rigid phase, as seen in Fig. 2D and Fig. S4A. This suggests that aerosolized LB will exhibit some particle-to-particle variation; some particles are more semi-solid than others. This may be due to a nucleation-initiated (and thus stochastic and time-dependent) aggregation or gelation process that leads to a finite probability of some particles remaining as a viscous semi-solid, as suggested by Fig. S5B. All of these morphologies are consistent with a semi-solid state, and this variability does not change the overall conclusions of the manuscript. Due to this variability, it is likely that viral viability reflects a statistical average of the outcomes in these different micro-environments. **(B)** Semi-solid DMEM coalescence at 50% RH. Initial coalescence was fast relative to our ability to image the process, as the initial aspect ratio after merging was ~1.67, indicating significant coalescence initially. After initial coalescence, the aspect ratio decreases more slowly and levels at an aspect ratio of 1.52 (after ~500 s), demonstrating a semi-solid state, likely a fluid and gel state coexisting. **(C)** TSB coalescence at 45% RH, where TSB began to show partial coalescence. Above 45% RH, complete coalescence was observed. When the final aspect ratio was ~1.1 or less, as in this example at 45% RH, we estimated the viscosity from the timescale of coalescence (τ = 262 s, viscosity of ~8×10^5 Pa·s). **(D)** 1:1 NaCl:protein coalescence at 70% RH to an aspect ratio of ~1.1 with a time constant of 74 s (viscosity ~2×10^5 Pa·s), demonstrating a viscous semi-solid state.

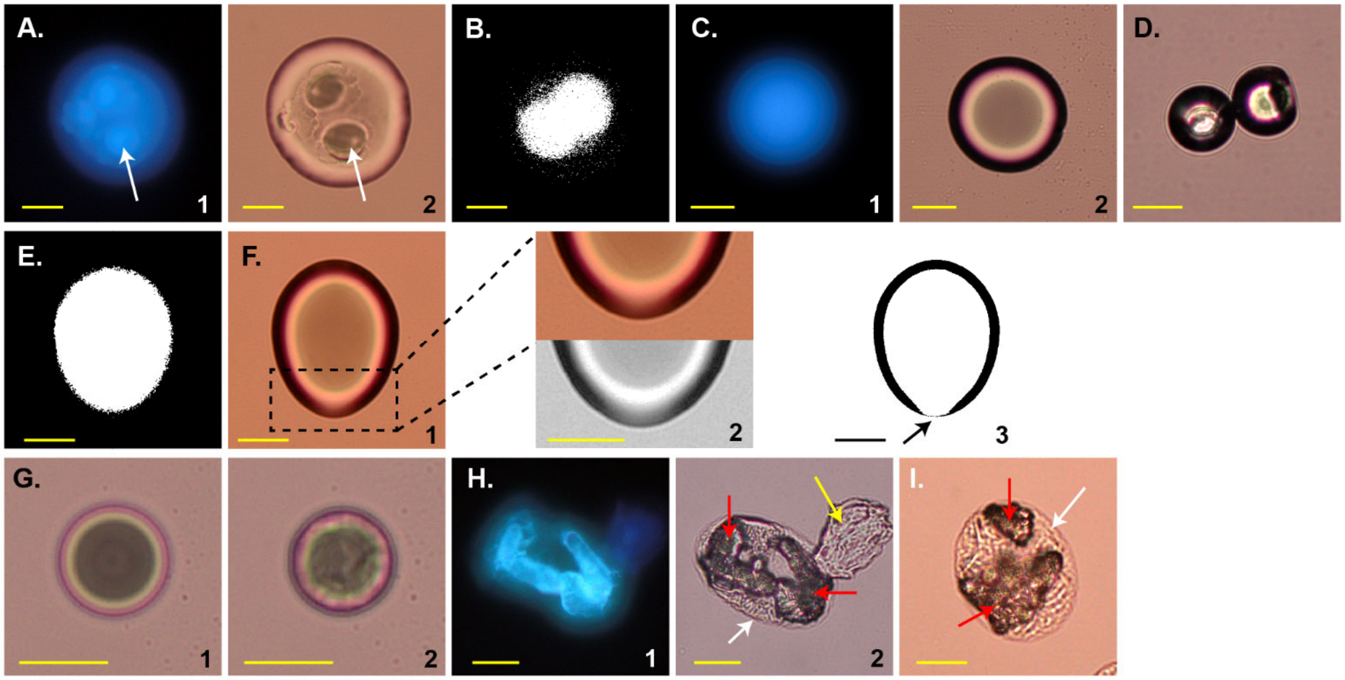

Figure S4. Images of merged aerosols that were ejected from the DBQ-EDB onto a microscope slide. All scale bars are 20 µm. **(A) LB** aerosols merged at 55±2% RH. Panel 1 shows the fluorescence image, and panel 2 shows the brightfield image. The dense, amorphous regions in the brightfield images are associated with higher fluorescence; an example is indicated by the arrow. This indicates the amorphous regions are enriched in organic compounds, possibly through aggregation or other phase separation. There are two organic-dense regions, presumably one from each of the aerosols that were merged. Ambient laboratory air during imaging was ~65% RH. **(B)** A binary contrast image of Fig. 2E, panel 1 from the main manuscript of merged DMEM aerosols. The threshold intensity was set to isolate the amorphous region, which had a higher fluorescence intensity than the surrounding fluid phase. A dumbbell shape is seen, indicating the two amorphous regions of each individual aerosol did not coalesce into a sphere, but did clump together. **(C) TSB** merged at 48±2% RH. The merged aerosols were observed to coalesce prior to ejecting onto the slide. Panel 1: fluorescence image. Panel 2: brightfield image. The images show an amorphous, homogeneous mixture. No changes to morphology were observed after blowing dry air over the particle. Ambient laboratory air during imaging was ~50% RH. **(D)** TSB merged at 35±4% RH and ejected ~10 s after merging (after collecting a brightfield image in the DBQ-EDB to confirm the dimer was rigid). The image shows a rigid dimer. The right side has been flattened and potentially fractured, likely due to impact with the substrate. **(E)** Binary contrast image of Fig. 3D, panel 1 for 1:1 NaCl:protein fluorescence. **(F)** Analysis of the image shown in Fig. 3D, panel 2, for 1:1 NaCl:protein. Here, panel 1 shows the brightfield image. Panel 2 focuses on an area where there are heterogeneities in contrast. Bottom shows the grayscale image (red color plane). Panel 3 shows a binary contrast image with a threshold intensity of 105, which further demonstrates uneven contrast and suggests a difference in refractive index, possibly due to a partially-engulfed morphology. Note that upon efflorescence, an NaCl crystal forms at the same location, as seen in Fig. 3D, panel 3 of the main text. **(G)** An example of merged 1:1 NaCl:albumin aerosols at 60±1% RH. In this example, the merged dimer is oriented vertically such that only the top particle is visible. Panel 1 shows the amorphous particle, and panel 2 shows efflorescence after blowing dry air over the slide. Although spherical aberrations obscure the image, it is seen that the effloresced particle remains mostly spherical with a roughened interior. Ambient laboratory air during imaging was ~55% RH. **(H)** 1:1:0.1 NaCl:protein:$CaCl_2$ merged at 75±3% RH. Two distinct salt crystals (red arrows) are observed surrounded by an amorphous phase (white arrow). In the brightfield, a piece of laboratory dust is also visible, as indicated by the yellow arrow in panel 2. This was assumed to settle onto the slide after collection of the merged aerosols. Ambient laboratory air during imaging was ~55% RH. **(I)** An additional example of 1:1:0.1 NaCl:protein:$CaCl_2$ aerosols merged at 75±3% RH. Two salt crystals are observed (red arrows) surrounded by an amorphous phase (white arrow). Ambient laboratory air during imaging was ~55% RH. In all instances of ejecting NaCl:protein:$CaCl_2$ aerosols from the DBQ-EDB, they were observed to have crystallized after collection on a substrate. For this composition, the impact on the substrate may initiate contact efflorescence.

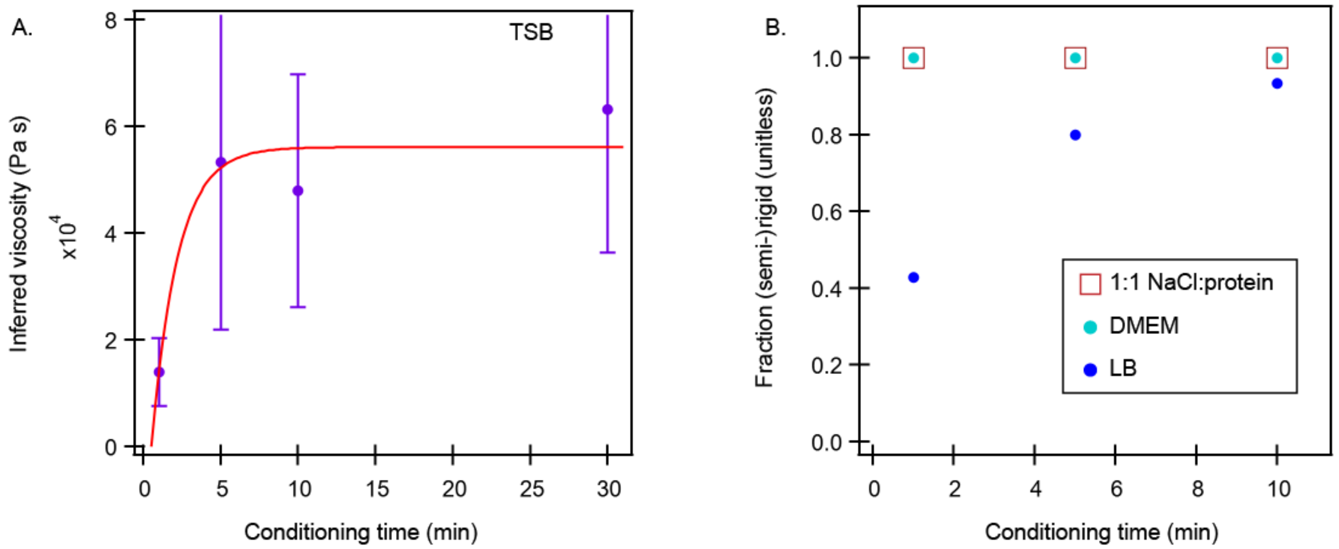

Figure S5. The phase state of aerosols as a function of pre-merging conditioning time. **(A)** The inferred viscosity of TSB aerosols as a function of time after initial trapping in the DBQ-EDB at 50±2% RH. Within 1 min, the viscosity is >10^4 Pa·s, demonstrating an increase in viscosity that is rapid relative to the lifetime of most viruses. The viscosity plateaus after ~5 min. The red line is an exponential fit to the data to visualize the time-dependent trend. The error bars are ±1 SD of at least 4 trials. The magnitude of the error bars is typical for viscosity measurements that are inferred through aerosol coalescence (1, 3). **(B)** The fraction of merged aerosols that were partially-rigid after conditioning times of 1, 5 and 10 min for DMEM, LB, and 1:1 NaCl:protein at 50±2, 50±2, and 65±3% RH, respectively. The fraction of partially-rigid aerosols was determined as the number of merging events that resulted in a dimer with a final aspect ratio greater than 1.2 divided by the total number of observed merging events. DMEM and NaCl:protein aerosols were partially-rigid at all conditioning times, suggesting the aerosols rapidly adopt the semi-solid state. For LB aerosols, nearly half were partially-rigid after 1 min, with a majority being partially-rigid after 5 min. This time-dependent behavior suggests a nucleation and growth mechanism. A minimum of seven trials were performed for each composition and conditioning time. To perform the experiments in A and B, aerosols were trapped in the bottom balance and the top balance, and then conditioned at a fixed RH for the set amount of time. The time between trapping in the bottom balance and top balance is ~15 sec. Thus, the conditioning of the particle in the bottom balance is slightly longer than the particle in the top balance. After conditioning, the aerosols were then merged and viscosity or aspect ratio was assessed. The aerosols used here are ~20 µm in diameter, which is larger than the typical respiratory aerosol. Ambient respiratory aerosols will thus equilibrate even more rapidly due to the smaller length scales.

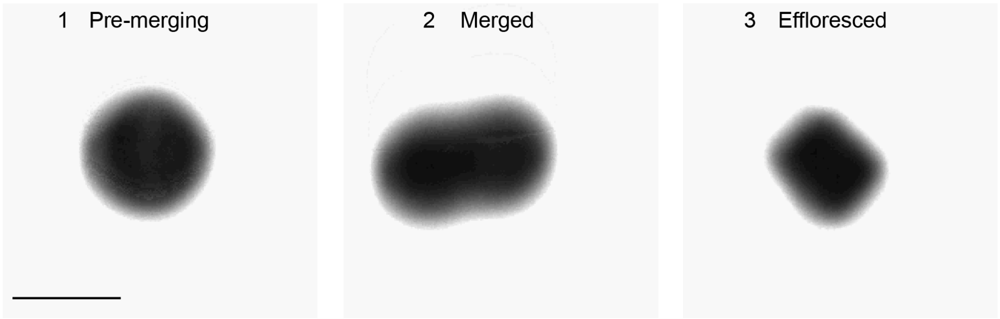

Figure S6. An example of contact-induced efflorescence of 1:1 NaCl:protein aerosols, initiated upon merging at 60±2% RH. Frame 1 shows a particle in the lower balance pre-merging. Frame 2 shows the merged dimer, ~2 seconds after merging. It is seen that they exist as a rigid dimer, indicating a viscous phase. However, the dimer began to lose mass and moved out of the field-of-view of the imaging camera. In frame 3, after bringing the particle back into the image, it is seen that the particles have effloresced and are in a cubic structure. It is likely that the particles exist in a phase separated morphology, with a viscous protein-enriched shell and an aqueous core. Merging the particles initiates nucleation, possibly through a similar mechanism as contact-induced efflorescence of NaCl initiated by polystyrene latex spheres, as reported in Davis and Tolbert (11). That is, the merging of the particles may cause some protein to mix with the aqueous core and destabilize the aqueous phase, initiating nucleation. Scale bar is 30 µm. In the absence of mucin, 1:1 NaCl:albumin aerosols do not effloresce upon contact.

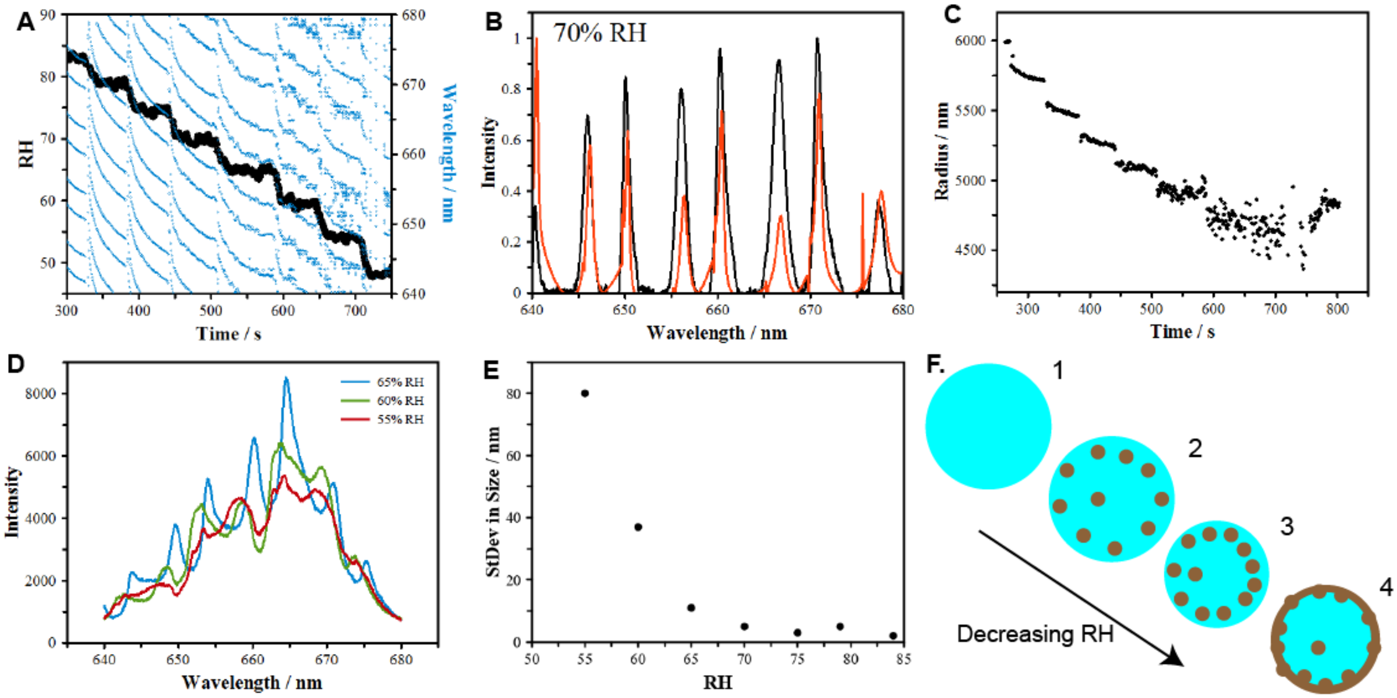

Figure S7. Results from Mie resonance spectra for singly levitated aerosol particles of 1:1 NaCl:protein. Mie resonance spectroscopy was used as a complementary approach to explore the phase state of NaCl-protein mixtures. The data shown in parts A-D are for the same particle. **(A)** A single particle was initially levitated at ~84% RH. Mie resonance spectra were collected as a function of time under variable RH conditions. The peak positions from the Mie spectra are plotted in blue, while the RH is plotted in black. A smooth change in peak position, as seen between 0 and ~450 s (RH ~84 and 70%), is indicative of a spherical particle. At longer timescales, there is significant variation in peak position, indicative of increasing disruptions in the Mie resonance spectra. **(B)** An example of a Mie resonance spectrum collected at 70% RH. Sizing is performed by comparing the experimental spectrum (black) to Mie theory predictions (red). In this spectrum, there is a good match between experiment and prediction, indicating a spherical particle and accurate sizing. **(C)** Extracted size as a function of time for the same particle discussed in A and B. At time <450 s (RH > 70%), the extracted size shows a smooth change in size (i.e., low variability between data points). At longer timescales (>450 s, RH<70%) there is an increase in the variability in the data, suggesting the particle is no longer homogeneously mixed. This increase in variability in extracted size may be due to protein aggregates or liquid-like protein nanodroplets that are disrupting the Mie resonances (28) . The spread in data increases with decreasing RH. **(D)** The source of the variability in the sizing data can be seen in the Mie spectra with decreasing RH. At ~65% RH, peaks are still sharp and fairly well-resolved, which is indicative of a mostly-spherical particle, but (as noted in part C) there is significant variation in the extracted size, likely due to protein aggregates or other protein-dense phase. By 60% RH, the structure is lost, demonstrating a non-homogeneous particle. **(E)** The standard deviation in size as a function of RH for a different 1:1 NaCl:protein particle than that discussed in parts A-D. Rather than continuously varying the RH, the RH was equilibrated and held at a constant RH, and then stepped to a lower RH. Under these conditions, it is also seen that particle sizing becomes increasingly uncertain below ~70% RH, which indicates loss of homogeneity. **(F)** The data discussed in parts A-E point toward the formation of heterogeneities within the particle at ~65-70% RH, which is coincident with the RH where coalescence measurements indicate a semi-solid state. These changes observed in the Mie resonance spectra are consistent with some form of phase separation. Initially (~70% RH), the disruptions in the Mie resonance are random. It is thus likely that protein aggregation (or formation of a protein-dense fluid region) is occurring, and those aggregates randomly disrupt the Mie resonance (28). Considering that the air-liquid interface can trigger protein aggregation (13), colloidal particles often have a tendency to partition to the air-liquid interface (29), and the enhancement of albumin at the air-liquid interface of salt solutions (30), it is likely that protein aggregates either form at the interface or partition to the interface after forming in the bulk, consistent with microscopy images showing a protein-enriched shell (Fig. 3D), although some aggregates likely remain in the bulk. The increasing disruption in the fringes (<65% RH) suggests the formation of more aggregates or aggregate coalescence and growth. The presence of highly distorted Mie resonances indicates a roughly spherical particle with significant heterogeneities (28). Considering the potential for the formation of protein aggregates at the interface, the Mie resonance data, coalescence measurements, and microscopy images, we propose the following

process for phase separation in NaCl-protein mixtures: protein aggregation or other phase separation (illustrated in panel 1 to 2), either at the air-liquid interface or within the particle bulk followed by surface partitioning; accumulation and compaction of the protein aggregates at the particle surface (illustrated in panel 2 to 3); and then coalescence or maturation into a protein-dense shell (illustrated in panels 3 to 4). Although the exact mechanism of phase separation is unclear, all data is consistent with the formation of a protein-enriched region of the particle.

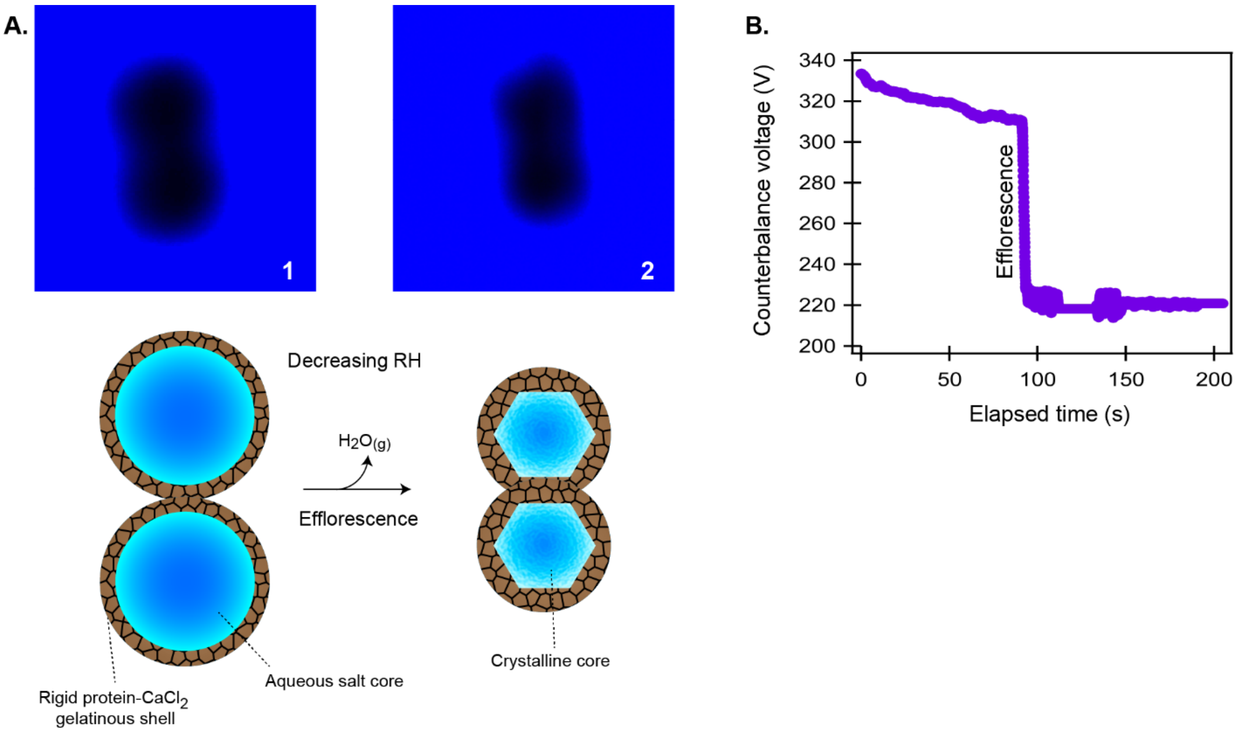

Figure S8. An example of efflorescence of rigid, merged aerosols of 1:1:0.1 NaCl:protein:$CaCl_2$, indicating a rigid gelatinous shell and aqueous core. **(A)** Bright-field images (top) and illustrated representation (bottom). Frame 1 shows the bright-field image of aerosols merged at 61±1% RH. The merged particles remained rigid, and did not coalesce. The RH was subsequently lowered from 61 to 44% RH. At 44±2% RH, the merged dimer abruptly lost mass and changed in morphology, although the dimer remained oblong. These changes are consistent with efflorescence of the aqueous NaCl. **(B)** The counterbalance voltage necessary to levitate the same dimer shown in part A as a function of time as the RH was lowered. At $t = 0$ s, the RH was ~48%. A gradual change in counterbalance voltage is seen as the particle loses water mass as RH decreases. At an elapsed time of ~90 s (which corresponded to an RH of ~44±2% RH), a rapid and abrupt change in counterbalance voltage (and thus particle mass) is seen, coincident with the morphological changes seen in part A. This is a clear indicator of efflorescence of the aqueous core, while the rigidity of the dimer (seen in the brightfield) is a clear indicator for a viscous, gelatinous shell that is sufficiently rigid to prevent coalescence. Further evidence for this morphology can be seen in Fig. S4H and S4I. This suggests that pathogens in respiratory particles can be in drastically different environments, depending on their location within the particle.

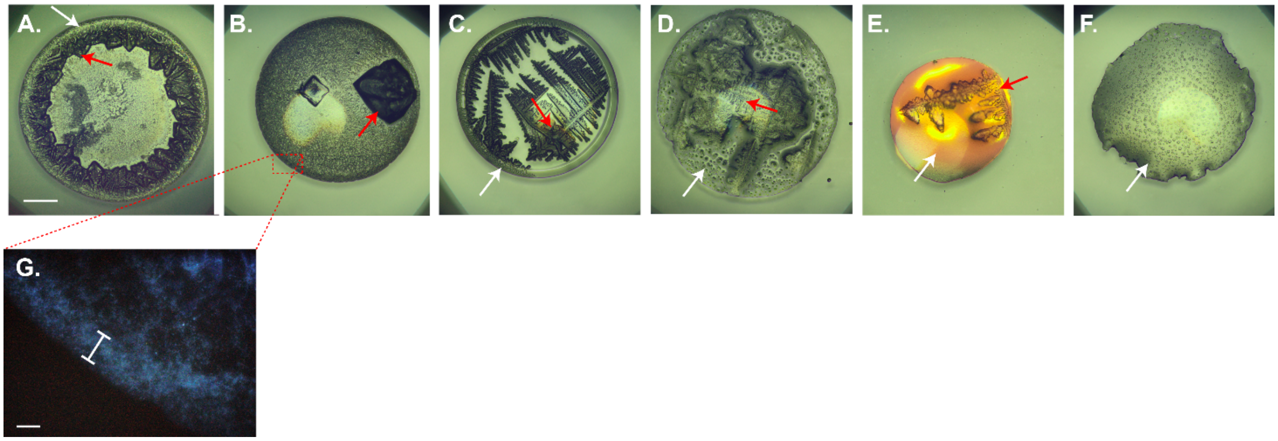

Figure S9. Microscopy images of droplets deposited on hydrophobic slides and exposed to laboratory air (~40% RH). **A-F** shows bright-field images (collected with episcopic illumination) corresponding to the fluorescence images shown in Fig. 4 of the main manuscript. **(A)** 1:1 NaCl:protein. **(B)** 10:1 NaCl:mucin. **(C)** 1:1:0.1 NaCl:protein:$CaCl_2$. **(D)** LB. **(E)** DMEM. **(F)** TSB. Scale bar in A-F (shown in part A) is 200 µm. Red arrows indicate crystalline phases. White arrows indicate amorphous phases. **(G)** Fluorescence microscope image (UV excitation) of the 10:1 NaCl:mucin droplet, imaged at 20X magnification. Although fluorescence was too weak to observe at 2.5X illumination (seen in Fig. 4B of the manuscript), higher magnification images suggest protein is concentrated at the edge of the drop, as indicated by the white line. Scale bar in part G is 25 µm. Contact angles are given in Table S2.

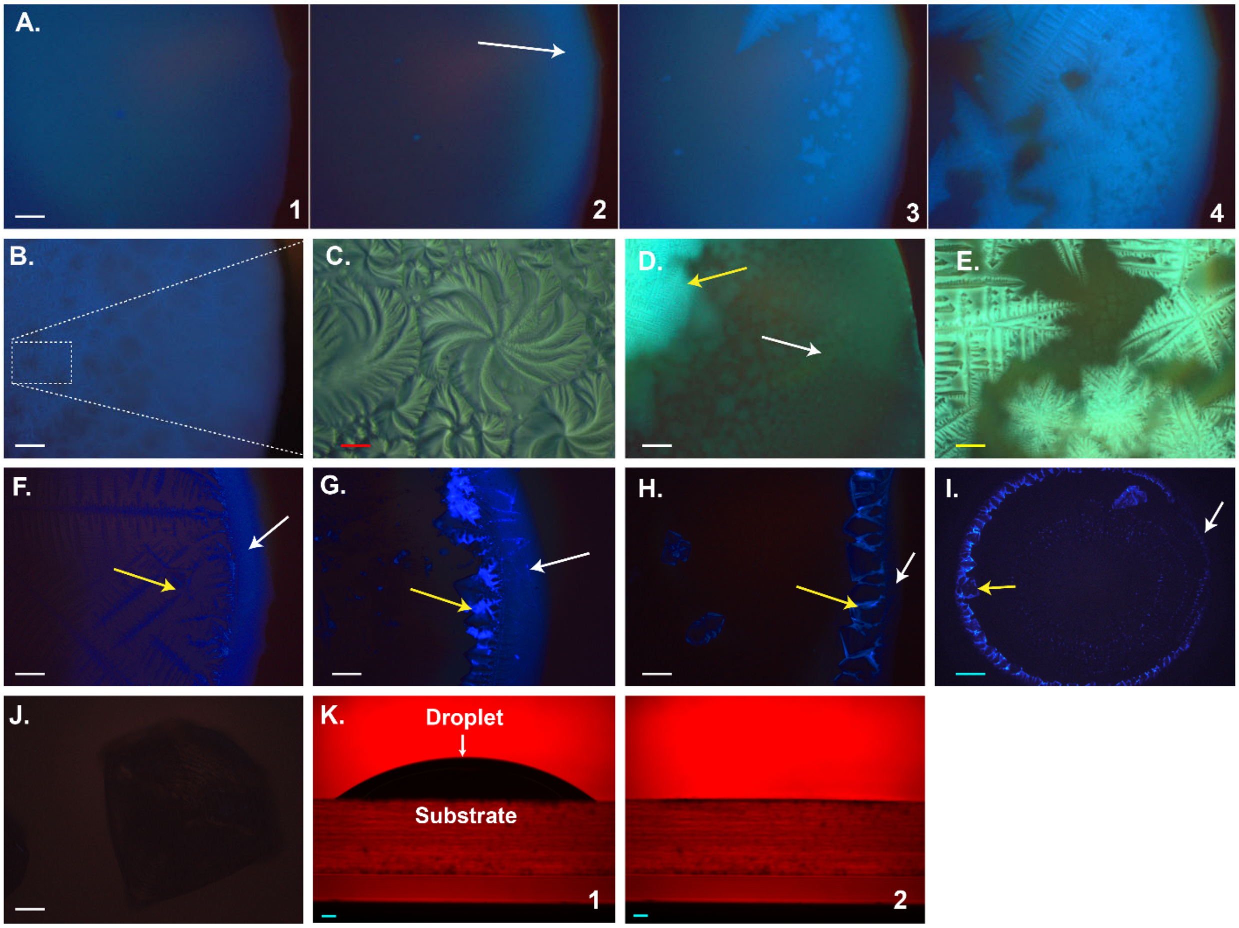

Figure S10. Microscopy images of droplets deposited on hydrophilic (untreated) microscope slides and exposed to laboratory air (~40% RH). **(A)** Time-dependent evaporation of LB droplets at 5X magnification and UV excitation. Immediately after droplet deposition (t = 0 s, frame 1) the fluorescent organic fraction is uniformly distributed across the droplet. As the droplet evaporates, some portion of the organic fraction accumulates at the edge of the droplet, evident by an increase in fluorescence at the edge of the drop and a decrease in the center of the droplet, as seen in frame 2 (see Fig. S11B for intensity profiles across the drop). At t = 568 s (frame 3), crystal nucleation and growth is evident. We note that the growing crystalline phase appears to have a higher fluorescence intensity than the surrounding solution. This is likely due to increased fluorescence intensity as a result of scattering and reflection of the excitation and emission wavelengths off of the irregular facets of the growing crystal. Although we cannot directly image the nucleation event, we can infer the general location of nucleation based on the progression of crystal growth. As seen in panel 3, there are nucleation sites that appear to be clustered ~100 µm from the edge of the droplet, potentially at the interface of the organic-dense region. Further, as seen at t = 840 s (frame 4), after crystal growth is complete, there is no visible presence of crystals along the outer edges of the droplet, presumably because the outer shell is primarily organic. This morphology is slightly different than for LB on a hydrophobic surface (Fig. 4D and S9D). **(B)** Evaporated TSB droplet (5X magnification, UV excitation), showing a mostly amorphous structure and uniform distribution of organic (relative to the salt-protein mixtures). There are some spiral structures visible, as shown in **(C)**, showing the spiral structures of TSB at higher magnification (20X, bright-field image). It is unclear what these structures are, but we note they were not observed in TSB deposited on a hydrophobic surface (Fig. 4F, S4C, S9F). **(D)** Evaporated DMEM droplet (5X magnification, UV excitation), showing a partially-crystalline phase within an amorphous phase. **(E)** Another image of the same DMEM droplet as in D, showing the center of the droplet at higher magnification (10X magnification). **(F)** 1:1:0.1 NaCl:protein:$CaCl_2$ (5X magnification, UV excitation). **(G)** 1:1 NaCl:protein (5X magnification, UV excitation). **(H)** 10:1 NaCl:mucin (5X magnification, UV excitation) **(I)** 10:1 NaCl:mucin (2.5X magnification, UV excitation). **(J)** NaCl (5X magnification, UV excitation) showing no fluorescence in the absence of protein/organic. **(K)** TSB evaporation viewed horizontally. Panel 1 shows the droplet shortly after deposition. The thickness of the droplet at the center is ~600 µm. Panel 2 shows the droplet after 30 min of evaporation. The droplet residue is <100 µm thick. White scale bar (A, B, D, F, G, H, J) is 100 µm. Red scale bar (C) is 25 µm. Cyan scale bars (I, K) are 200 µm. Yellow scale bar (E) is 50 µm. Contact angles are provided in Table S2.

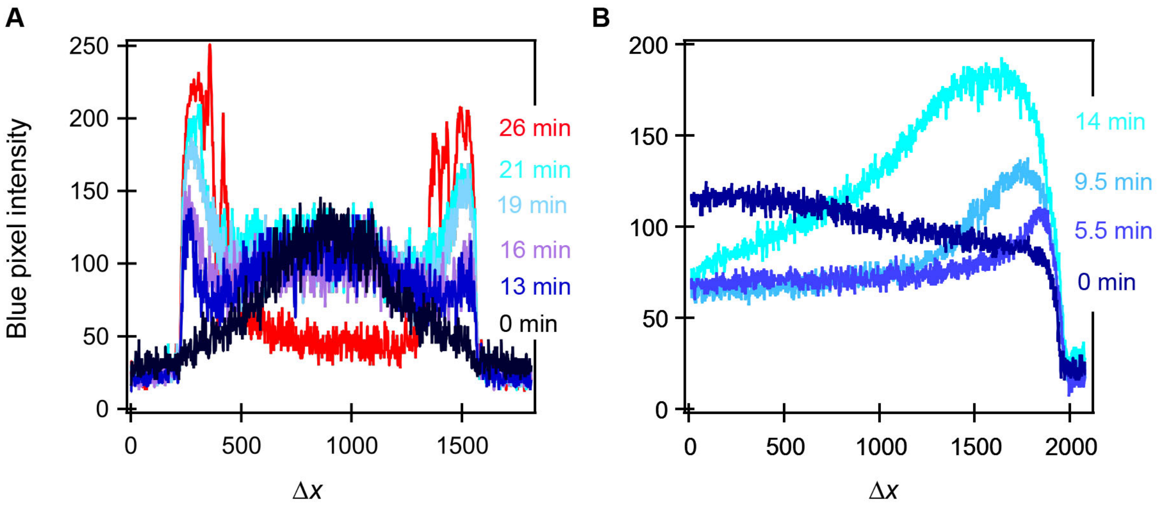

Figure S11. Blue pixel intensity profiles of evaporating droplets under UV excitation. Intensity was measured horizontally across images. **(A)** Intensity profiles for 1:1 NaCl:protein, corresponding to images in Fig. 4A of the main article. The top trace (black) is the initial frame ($t \approx 0$ s). Comparing the initial intensity distribution to final intensity distribution (final trace, red) shows that the organic (protein) fraction phase separates from the inorganic phase. **(B)** Intensity profiles for LB on a hydrophilic surface, corresponding to the image sequence shown in Fig. S10A. Only a portion of the droplet was captured in the image, with the center of the droplet on the left edge of the image ($\Delta x = 0$) and the right edge of the droplet on the right side of the image ($\Delta x \approx 2000$). There is clear accumulation of organic material at the outer edge of the droplet as evaporation proceeds.

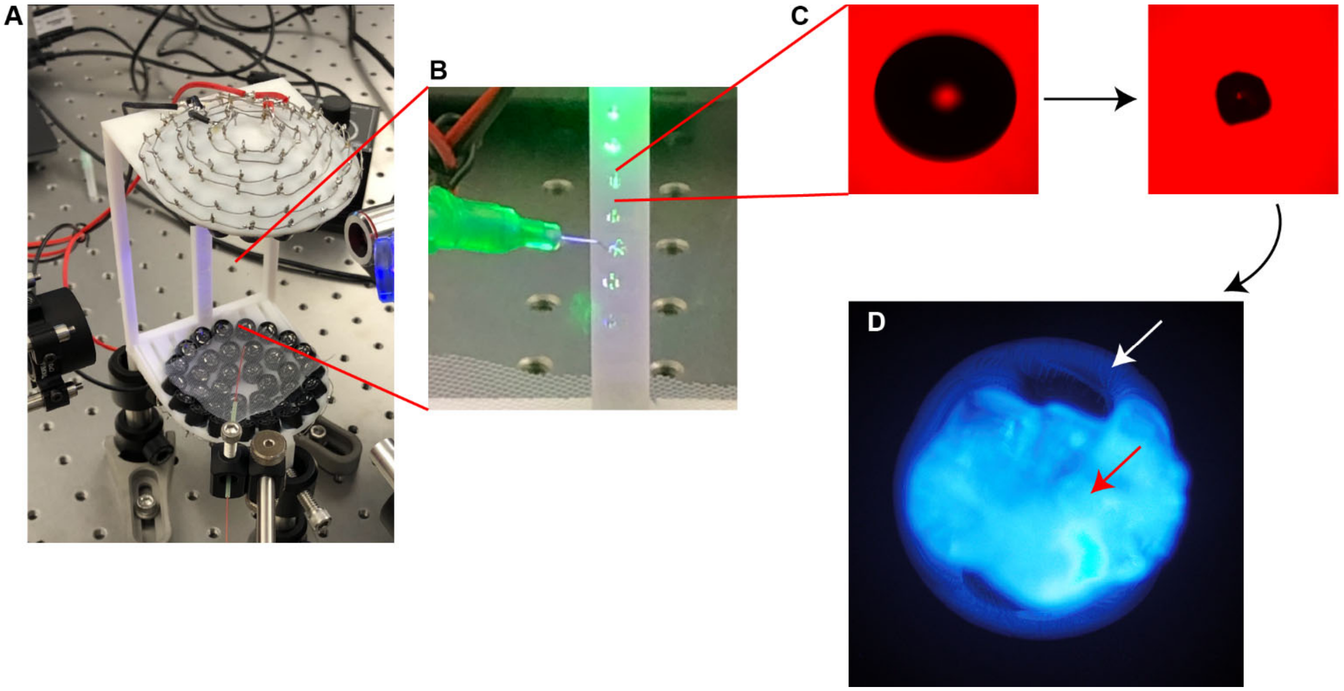

Figure S12. Phase separation in acoustically-levitated droplets of 1:1 NaCl:protein. In this experimental arrangement, there is no substrate during the evaporation phase. It is thus possible to test whether the phase-separated morphology observed in Fig. 4A of the manuscript is due to a substrate effect. Here, we levitated droplets until efflorescence, and then collected the effloresced particles on a microscope slide for fluorescence microscopy. **(A)** Droplets were acoustically-levitated in a multi-emitter setup that was constructed following the design and instructions provided in Marzo et al. (31). Despite being levitated in air, we still refer to these as "droplets" since they would rapidly fall under the force of gravity without the acoustic waves. **(B)** Multiple droplets, ~3 µL in volume, were loaded into the trapping site with a syringe. Multiple droplets were simultaneously levitated because it was found that collection on a microscope slide was difficult; a majority of particles bounced off the collection substrate and were lost. Levitating multiple droplets increased the chances of a successful collection. A green laser (<5 mW) was directed at the trapping site to assist in loading droplets at the proper location. The laser was turned off during the evaporation period. **(C)** Bright-field images were collected of levitated droplets. The camera was on a translation stage that allowed multiple droplets to be imaged by vertically translating the stage, although only one droplet could be imaged at a time. In the image shown, the time between trapping and efflorescence was 28 min at ~50% RH (which was the RH of the ambient laboratory air). Efflorescence in the large droplets was possible at a slightly higher RH than with aerosols due to the larger volume and increased probability for heterogeneous efflorescence. **(D)** Droplets collected onto substrates and imaged with UV fluorescence show a phase-separated morphology with a protein shell surrounding a crystalline core, as indicated by the white and red arrows, respectively. (As noted previously, it is likely that the crystalline core appears to have a higher fluorescence intensity because it is coated with protein. The crystalline core amplifies the fluorescence of the protein through reflection and scattering of the excitation and emission wavelengths.) This observation is consistent with all other methods of analysis, which indicate a phase separated morphology with NaCl-protein mixtures.

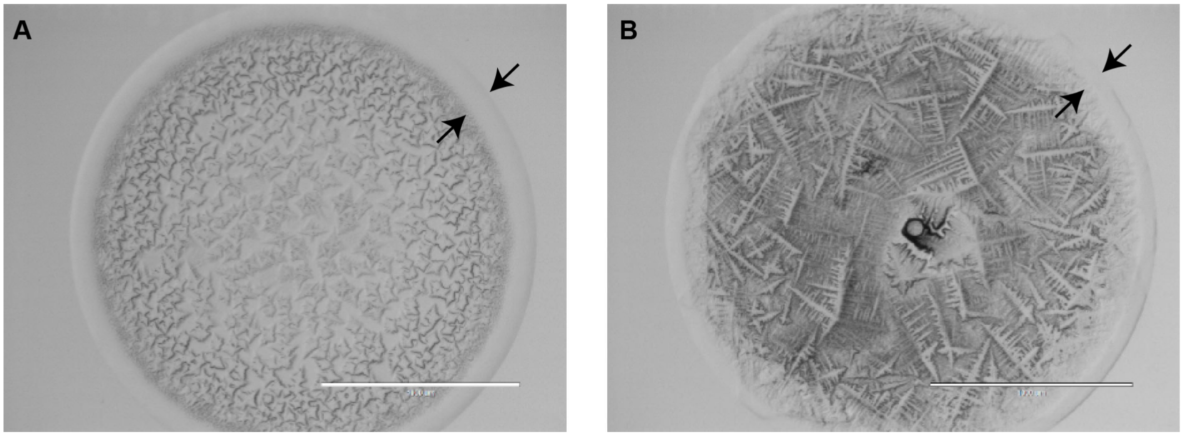

Figure S13. Morphology of evaporated LB droplets on a hydrophilic surface **(A)** without *E. coli*, and **(B)** with *E. coli*. As indicated by the arrows, there is an amorphous shell in both cases, suggesting that growth of pathogens within culture media will not change our reported observations of amorphous phase states, although the hygroscopicity may be altered to some extent. The crystal habit does change with the inclusion of *E. coli*. Although the NaCl crystal habit is changed, this would not necessarily indicate a change in efflorescence RH since moderate changes to solution composition can change the crystal habit of NaCl without changing the efflorescence RH (for example, compare the efflorescence RH of the NaCl-protein mixtures, which display a range of crystal habits while maintaining a consistent efflorescence RH of ~45%). Scale bar is 1000 µm.

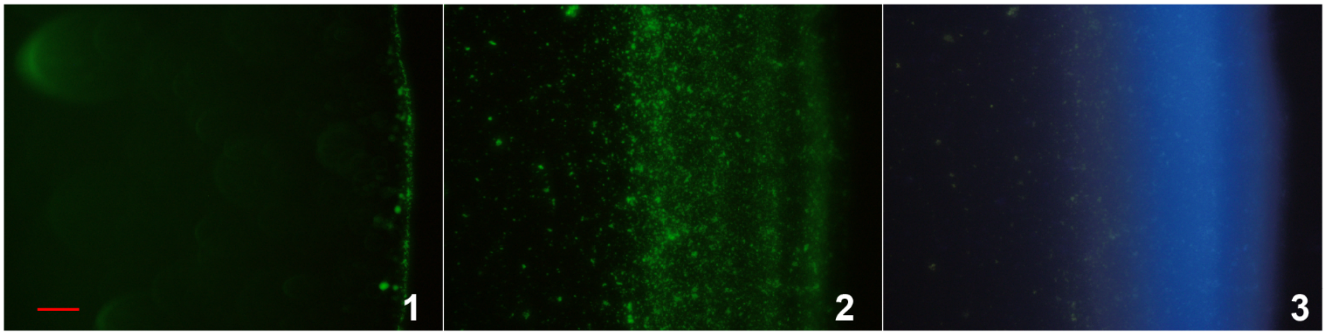

Figure S14. 1:1 NaCl:protein doped with 100 nm fluorescent PSLs, 20X magnification. Frame 1, t ≈ 0 s. Frames 2 and 3, t ≈ 10 min. Frame 2 shows the accumulation of the PSLs with blue excitation. Frame 3 shows with UV excitation that PSLs are accumulated with protein on the outer edge of the drop. Scale bar is 25 µm.

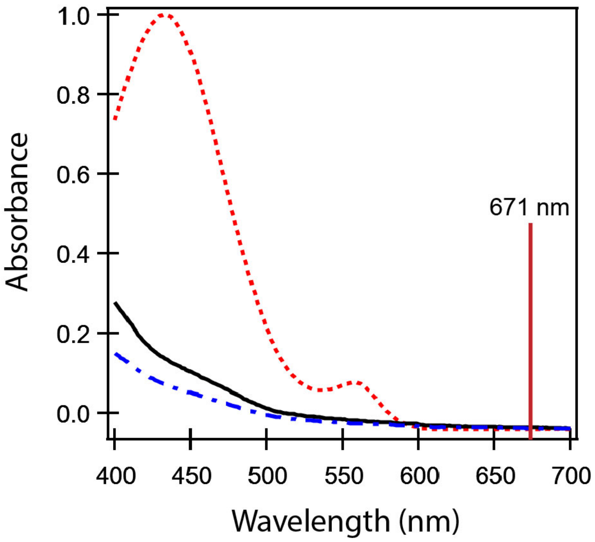

Figure S15. Visible absorbance spectra of the three culture media. Dotted red line: DMEM. Solid black line: TSB. Dash-dot blue line: LB. The laser line used for laser scatter imaging is indicated at 671 nm, demonstrating this wavelength does not correspond to any absorption features associated with the culture media.

Table S1. The composition of the growth media

Dulbecco's modified Eagle's medium[1]		Lysogeny broth (Miller)[2]		Tryptic soy broth[3]	
**Component**	**Dry mass fraction**	**Component**	**Dry mass fraction**	**Component**	**Dry mass fraction**
Sodium Chloride	0.479	Sodium chloride	0.4	Sodium chloride	0.167
Calcium Chloride	1.50E-02	Tryptone	0.4	Peptone (from casein digest)	0.567
Ferric Nitrate • 9H2O	7.49E-06	Yeast extract	0.2	Peptone (from soymeal digest)	0.100
Magnesium Sulfate	7.31E-03			Dipotassium phosphate	0.083
Potassium Chloride	2.99E-02			D-glucose	0.083
Sodium Phosphate Monobasic	8.16E-03				
**Inorganic salts (total):**	**0.539**				
L-Arginine • HCl	6.29E-03				
L-Cystine • 2HCl	4.69E-03				
L-Glutamine	4.37E-02				
Glycine	2.25E-03				
L-Histidine • HCl • H2O	3.14E-03				
L-Isoleucine	7.86E-03				
L-Leucine	7.86E-03				
L-Lysine • HCl	1.09E-02				
L-Methionine	2.25E-03				
L-Phenylalanine	4.94E-03				
L-Serine	3.14E-03				
L-Threonine	7.11E-03				
L-Tryptophan	1.20E-03				
L-Tyrosine • 2Na • 2H2O	7.77E-03				
L-Valine	7.04E-03				
**Amino Acids (total)**	**0.120**				
Choline Chloride	2.99E-04				
Folic Acid	2.99E-04				
myo-Inositol	5.39E-04				
Niacinamide	2.99E-04				
D-Pantothenic Acid	2.99E-04				
Pyridoxal • HCl	2.99E-04				
Riboflavin	2.99E-05				
Thiamine • HCl	2.99E-04				
D-Glucose	0.337				
Phenol Red • Na	1.19E-03				

[1]Sigma-Aldrich product number D5648. [2]Sigma-Aldrich product number L3522. [3]Millipore product number 1054590500

Table 2. Contact angles of 3 µL droplets deposited on glass slides

Composition	Untreated glass (hydrophilic) Initial angle (degrees)	Treated glass (hydrophobic) Initial angle (degrees)
1:1 NaCl:protein	43±1	52±3
10:1 NaCl:mucin	15±2	55±3
1:1:0.1 NaCl:protein:$CaCl_2$	29±4	42±3
DMEM	26±3	41±3
TSB	27±3	41±2
LB	23±3	40±3

Caption for Movie S1. Shown is a movie of a levitated LB aerosol particle (at ~61% RH) illuminated with a 671 nm laser, and the elastically scattered laser light is imaged in the far-field, where the linear interference fringes are consistent with expectations for a liquid aerosol particle. There are subtle non-uniformities, evident as an increased brightness and disruption in the linear fringes, that indicate a slight inhomogeneity in the aerosol particle. This is likely due to aggregation or liquid-liquid phase separation where the outer organic shell is not uniformly distributed (possibly as a partially-engulfed configuration). Similar fluctuations were observed in 1:1 NaCl:protein aerosol at >70% RH, 10:1 NaCl:mucin aerosol at ~60% RH, and 1:1:0.1 NaCl:protein:$CaCl_2$ aerosol at >85% RH. These systems likely undergo some form of phase separation, which is likely the source of the non-uniformities in the far-field fringes as light propagates through regions with different refractive indices.

Supplemental References

1. D. S. Richards, K. L. Trobaugh, J. Hajek-Herrera, R. D. Davis, Dual-Balance Electrodynamic Trap as a Microanalytical Tool for Identifying Gel Transitions and Viscous Properties of Levitated Aerosol Particles. *Anal. Chem.* **92**, 3086–3094 (2020).
2. D. R. Absolom, C. J. Van Oss, W. Zingg, A. W. Neumann, Determination of surface tensions of proteins II. Surface tension of serum albumin, altered at the protein-air interface. *Biochim. Biophys. Acta - Protein Struct.* **670**, 74–78 (1981).
3. Y. C. Song, *et al.*, Measurements and Predictions of Binary Component Aerosol Particle Viscosity. *J. Phys. Chem. A* **120**, 8123–8137 (2016).
4. T. A. Wani, A. H. Bakheit, S. Zargar, M. A. Hamidaddin, I. A. Darwish, Spectrophotometric and molecular modelling studies on in vitro interaction of tyrosine kinase inhibitor Linifanib with bovine serum albumin. *PLoS One* **12**, 1–12 (2017).
5. C. L. Price, A. Bain, B. J. Wallace, T. C. Preston, J. F. Davies, Simultaneous Retrieval of the Size and Refractive Index of Suspended Droplets in a Linear Quadrupole Electrodynamic Balance. *J. Phys. Chem. A* **124**, 1811–1820 (2020).
6. D. J. Stewart, *et al.*, Liquid-liquid phase separation in mixed organic/inorganic single aqueous aerosol droplets. *J. Phys. Chem. A* **119**, 4177–4190 (2015).
7. D. S. Richards, *et al.*, Ion-molecule interactions enable unexpected phase transitions in organic-inorganic aerosol. *Sci. Adv.* **6**, eabb5643 (2020).
8. A. C. Dumetz, A. M. Chockla, E. W. Kaler, A. M. Lenhoff, Protein phase behavior in aqueous solutions: Crystallization, liquid-liquid phase separation, gels, and aggregates. *Biophys. J.* **94**, 570–583 (2008).
9. T. Koop, J. Bookhold, M. Shiraiwa, U. Pöschl, Glass transition and phase state of organic compounds: Dependency on molecular properties and implications for secondary organic aerosols in the atmosphere. *Phys. Chem. Chem. Phys.* **13**, 19238–19255 (2011).
10. S. T. Martin, Phase transitions of aqueous atmospheric particles. *Chem. Rev.* **100**, 3403–3453 (2000).
11. R. D. Davis, M. A. Tolbert, Crystal nucleation initiated by transient ion-surface interactions at aerosol interfaces. *Sci. Adv.* **3**, e1700425 (2017).
12. R. E. H. Miles, J. F. Davies, J. P. Reid, The influence of the surface composition of mixed monolayer films

on the evaporation coefficient of water. *Phys. Chem. Chem. Phys.* **18**, 19847–19858 (2016).

13. E. Koepf, S. Eisele, R. Schroeder, G. Brezesinski, W. Friess, Notorious but not understood: How liquid-air interfacial stress triggers protein aggregation. *Int. J. Pharm.* **537**, 202–212 (2018).
14. H. A. Kramers, Brownian motion in a field of force and the diffusion model of chemical reactions. *Physica* **7**, 284–304 (1940).
15. J. F. Davies, A. E. Haddrell, R. E. H. Miles, C. R. Bull, J. P. Reid, Bulk, surface, and gas-phase limited water transport in aerosol. *J. Phys. Chem. A* **116**, 10987–10998 (2012).
16. G. Gadda, P. Sobrado, Kinetic Solvent Viscosity Effects as Probes for Studying the Mechanisms of Enzyme Action. *Biochemistry* **57**, 3445–3453 (2018).
17. S. F. Scarlata, "The Effects of Increased Viscosity on the Function of Integral Membrane Proteins" in *High Pressure Effects in Molecular Biophysics and Enzymology*, (1996), pp. 331–345.
18. K. Lin, L. C. Marr, Humidity-Dependent Decay of Viruses, but Not Bacteria, in Aerosols and Droplets Follows Disinfection Kinetics. *Environ. Sci. Technol.* **54**, 1024–1032 (2020).
19. M. Shiraiwa, M. Ammann, T. Koop, U. Pöschl, Gas uptake and chemical aging of semisolid organic aerosol particles. *Proc. Natl. Acad. Sci. U. S. A.* **108**, 11003–11008 (2011).
20. A. J. Prussin, *et al.*, Survival of the enveloped virus Phi6 in droplets as a function of relative humidity, absolute humidity, and temperature. *Appl. Environ. Microbiol.* **84**, 1–10 (2018).
21. D. H. Morris, *et al.*, Mechanistic theory predicts the effects of temperature and humidity on inactivation of SARS-CoV-2 and other enveloped viruses. *Elife* **10** (2021).
22. T. Koop, A. Kapilashrami, L. T. Molina, M. J. Molina, Phase transitions of sea-salt/water mixtures at low temperatures: Implications for ozone chemistry in the polar marine boundary layer. *J. Geophys. Res. Atmos.* **105**, 26393–26402 (2000).
23. I. V. Polozov, L. Bezrukov, K. Gawrisch, J. Zimmerberg, Progressive ordering with decreasing temperature of the phospholipids of influenza virus. *Nat. Chem. Biol.* **4**, 248–255 (2008).
24. B. B. Hasinoff, S. B. Chishti, Viscosity Dependence of the Kinetics of the Diffusion-Controlled Reaction of Carbon Monoxide and Myoglobin. *Biochemistry* **21**, 4275–4278 (1982).
25. V. H. Carvalho-Silva, N. D. Coutinho, V. Aquilanti, Temperature dependence of rate processes beyond Arrhenius and eyring: Activation and transitivity. *Front. Chem.* **7** (2019).
26. A. Panagopoulou, *et al.*, Glass transition and dynamics in BSA-water mixtures over wide ranges of composition studied by thermal and dielectric techniques. *Biochim. Biophys. Acta - Proteins Proteomics* **1814**, 1984–1996 (2011).
27. B. Zobrist, *et al.*, Ultra-slow water diffusion in aqueous sucrose glasses. *Phys. Chem. Chem. Phys.* **13**, 3514–3526 (2011).
28. M. I. Mishchenko, L. Liu, D. W. Mackowski, Morphology-dependent resonances of spherical droplets with numerous microscopic inclusions. *Opt. Lett.* **39**, 1701 (2014).
29. D. F. Williams, J. C. Berg, The aggregation of colloidal particles at the air—water interface. *J. Colloid Interface Sci.* **152**, 218–229 (1992).
30. Y. Li, *et al.*, Salting up of Proteins at the Air/Water Interface. *Langmuir* **35**, 13815–13820 (2019).
31. A. Marzo, A. Barnes, B. W. Drinkwater, TinyLev: A multi-emitter single-axis acoustic levitator. *Rev. Sci. Instrum.* **88** (2017).
